# Mobilization of Unconsolidated Granular Material on Asteroid (101955) Bennu by Spacecraft Interaction

**DOI:** 10.1007/s11214-026-01285-8

**Published:** 2026-04-01

**Authors:** Edward B. Bierhaus, Jarvis T. Songer, Courtney E. Mario, Christopher D. Norman, Curtis Miller, Ryan Olds, Angelica Martinez, Benton C. Clark, Christine Hartzell, Bashar Rizk, Christian Drouet d’Aubigny, Jennifer Nolau, Alicia Allen, Maurizio Pajola, Dathon R. Golish, Humberto Campins, Kevin J. Walsh, Ronald-Louis Ballouz, C. W. V. Wolner, Brent J. Bos, Dante S. Lauretta, Michael C. Nolan, Daniella N. DellaGiustina

**Affiliations:** 1 Lockheed Martin Space, Littleton, CO USA; 2 Draper, Cambridge, MA USA; 3 Space Science Institute, Boulder, CO USA; 4 University of Maryland, College Park, MD USA; 5 Lunar and Planetary Laboratory, University of Arizona, Tucson, AZ USA; 6 University of Central Florida, Orlando, FL USA; 7 INAF – Astronomical Observatory of Padova, Padova, Italy; 8 Southwest Research Institute, Boulder, CO USA; 9 Applied Physics Laboratory, John Hopkins University, Laurel, MD USA; 10 NASA Goddard Spaceflight Center, Greenbelt, MD USA

**Keywords:** OSIRIS-REx, Asteroid, Regolith, Spacecraft thrusters, Sample collection

## Abstract

NASA’s OSIRIS-REx mission demonstrated the potential for robotic spacecraft to probe physical properties of planetary bodies. In 2020, the spacecraft autonomously collected granular material from Bennu, a small unconsolidated asteroid, leaving behind a region excavated by the combined effects of the gas-driven sampler and backaway thrusters. Disambiguating the physical responses to these two energy-injection events offers an opportunity to characterize a microgravity asteroid’s near-surface properties and understand how thruster-surface interactions could be utilized by future missions. We do so here using data from the spacecraft’s instruments and telemetry in conjunction with detailed modeling of the thrusters. The sampler initially formed a crater 0.5–0.7 meters in radius before thruster activation. The thrusters deposited four regions of high-pressure gas ∼1–6 meters from the contact location, with lower pressures inside and beyond this region. As a result, the crater expanded into a region undergoing active erosion from the thrusters, and its final dimensions were increased by thruster effects. The total erosion and redeposition depend on the pre-existing mass distribution and topography of the area. Varying erosion responses to the thrusters indicate variability in material properties laterally, and, combined with accelerometer data, as a function of depth. The efficacy of the thrusters to mobilize material over a broad area (>100 square meters), and at very small pressures (perhaps as low as 0.005 Pa), motivates their use to interrogate small-body surface properties, particularly in the spacecraft’s planned 2029 encounter with asteroid Apophis.

**Supplementary Information:**

The online version contains supplementary material available at 10.1007/s11214-026-01285-8.

## Introduction

Rubble-pile asteroids are microgravity aggregates, a physical structure unlike larger planetary bodies (Farinella et al. 1982; Scheeres et al. 2010; Walsh 2018). The particle sizes where non-gravitational forces exceed gravitational forces can occur at larger diameters than on terrestrial planets and the Moon (Scheeres et al. 2010; Hartzell and Scheeres 2011). This can lead to physical structures, or responses to external forces, that are unexpected from terrestrial geology. Some rubble piles originated in the early solar system by planetesimal formation processes that brought together centimeter-sized pebbles within the solar nebula (Nesvorný et al. 2021), whereas others are the byproduct of collisional fragmentation that has occurred throughout solar system history, with ejected debris agglomerating into rubble-pile structures. These gravitational aggregates are among the most numerous bodies of the solar system, especially in near-Earth space, motivating the derivation of accurate physical properties.

Robotic spacecraft have probed the physical construction of four rubble-pile asteroids, each <1 km in longest dimension: Japan’s Hayabusa mission bounced against Itokawa (Fujiwara et al. 2006); the derived coefficient of restitution was ∼0.84, larger than measured for granular material in microgravity (Yano et al. 2006). Hayabusa2 fired a projectile into Ryugu (Watanabe et al. 2019); the resulting crater indicated a nearly strengthless surface (Arakawa et al. 2020). The deliberate impact of NASA’s DART (Double Asteroid Redirection Test, Rivkin et al. 2021) spacecraft into Didymos demonstrated a similar lack of cohesive strength (Raducan et al. 2024).

NASA’s OSIRIS-REx (Origins, Spectral Interpretation, Resource Identification, and Security–Regolith Explorer) spacecraft used a gas-driven technique (Clark et al. 2016; Bierhaus et al. 2018) to collect unconsolidated granular material (regolith) on asteroid Bennu (Lauretta et al. 2017, 2022). The spacecraft’s Touch-and-Go Sample Acquisition Mechanism (TAGSAM) extended on a 3-m-long arm and fired nitrogen gas into the surface to mobilize regolith into a collection chamber (Clark et al. 2016; Bierhaus et al. 2018); just 6 s after contact, the backaway thrusters fired to move the spacecraft away from the asteroid. The short contact time, without “landing” the spacecraft on a microgravity surface, motivated the nickname Touch-and-Go, or TAG, for the sample activity. Evaluation of the spacecraft deceleration between surface contact and TAGSAM gas release (Walsh et al. 2022) and of Bennu’s surface after sampling (Lauretta et al. 2022) indicated that the surface regolith consists of loosely packed particles with little cohesion.

The total excavation by the spacecraft represents the combined effects of energy injected by TAGSAM and the thrusters into an unconsolidated microgravity surface. In this study, we use several data sources to disambiguate the relative effects of these gas-firing events and investigate the implications for Bennu’s near-surface properties: (i) sampling-event telemetry to define stages of the sampling sequence and the corresponding energy deposition into the regolith as a function of time and location; (ii) detailed modeling of the thruster plume interactions with the surface; and (iii) image data that enable measurements of material motion. Our analysis provides constraints on material properties with depth and across a broad lateral extent beyond the contact point.

Although engineers and scientists have recognized that spacecraft thrusters affect planetary surfaces since the Apollo era (Hutton 1968; Land and Scholl 1969), evaluation of thruster effects on planetary surfaces is often framed in the context of the potential risk to other spaceflight hardware (Metzger 2024a,b) or science measurements (Lorenz 2016). The OSIRIS-REx sampling event highlights that thruster-surface interactions provide a means to evaluate surface properties. Thus, in the case of low-gravity bodies where hovering and backaway are possible, surface properties can be assessed without direct contact, eliminating significant material risk and operational complexity when those properties are poorly constrained.

In 2029, the OSIRIS-REx spacecraft — now renamed OSIRIS–Apophis Explorer, or APEX — will encounter another rubble-pile asteroid, Apophis (DellaGiustina et al. 2023). Although TAGSAM is not available (the sampling device returned to Earth in 2023 with the collected sample, Bierhaus et al. 2018; Lauretta et al. 2024), a descent-and-backaway maneuver offers the possibility of manipulating the regolith via thrusters for scientific investigation of physical structure and space-weathering effects.

## The TAG Event

We use data that span the duration of the TAG sample-collection event to reconstruct an approximately chronological response of Bennu’s surface and subsurface to the gas injection events, though some analysis depends on data acquired later in time.

### The State of the Surface Before Spacecraft Interaction

The OSIRIS-REx sampling site resides inside a ∼20-m-diameter crater named Hokioi (Barnouin et al. 2022; Lauretta et al. 2022) with a relatively young estimated age (less than ∼2×10^5^ years, DellaGiustina et al. 2020; Bierhaus et al. 2023). The pre-existing surface and 3D distribution of mass at this location determine the source volumes and geometry of material that could be mobilized by the spacecraft, the regions available to unobstructed ballistic flow of mobilized ejecta, and regions that present obstacles to either ejected material or flow of the thruster gas across the topography of surface.

**Fig. 1:**
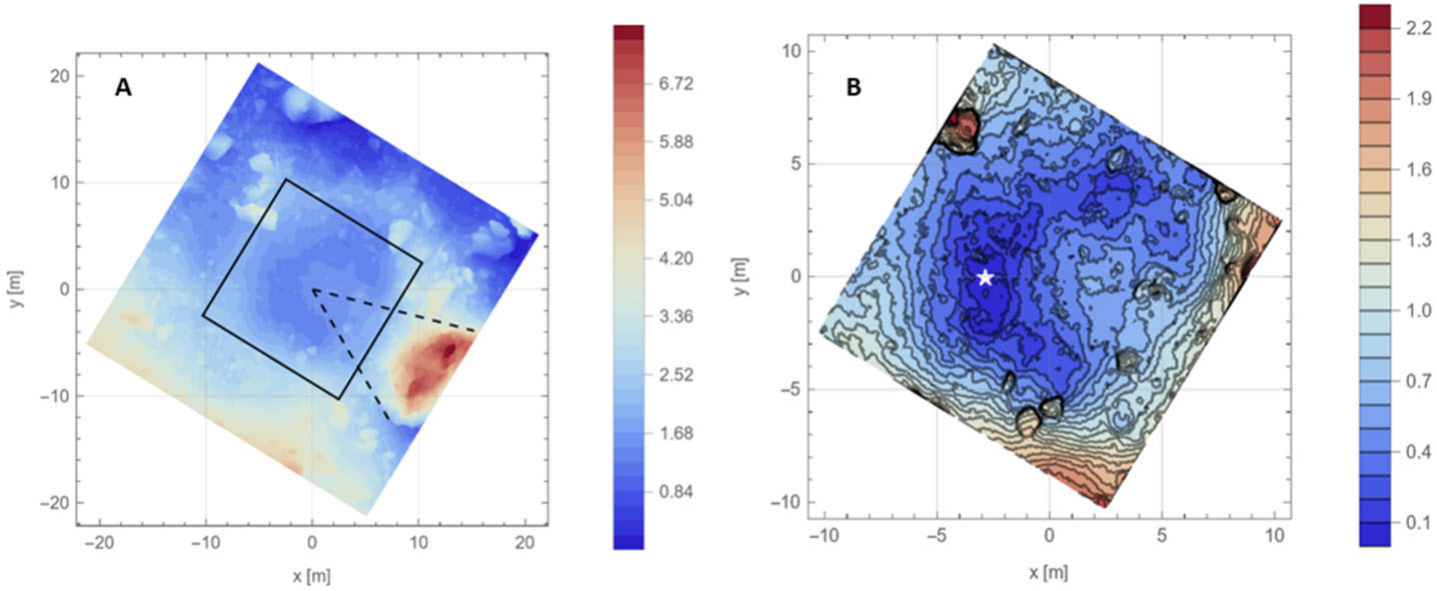
The pre-sampling surface (Lauretta et al. 2022); the TAG point is centered at (0, 0), and north is up. **(A)** Hokioi crater and surrounding terrain. Height is defined relative to the lowest location. The tall boulder to the southeast is informally called “Mt. Doom”. The black rotated square is the region shown in (B). The two dashed lines illustrate that the raised region inside Hokioi is in the direction of Mt. Doom. **(B)** A higher-resolution view of the boxed region from (A), with height contour lines every 10 cm. The white star is the lowest location in the DTM and defines zero height

The location of initial TAGSAM contact (the “TAG point”) is near the crater’s center. Considering height of the digital terrain model (DTM) of Hokioi crater from Lauretta et al. (2022) in a geometric sense in 3D space, and using the lowest part of Hokioi crater as a reference zero height, the TAG point is raised relative to most of its immediate surroundings (Fig. 1); within a radius of ∼4–5 m, only the region to the east/southeast is taller than the TAG point. Further from the TAG point, the Hokioi crater walls have typical rim heights of ∼1.8–2.3 m. Two exceptions are a group of rocks to the north/northwest with a maximum height of ∼4.2 m and a large boulder, informally named “Mt. Doom”, to the southeast with a maximum height of ∼7.5 m (Fig. 1A). Mt. Doom lies in the same direction as the increase in height of the surface from the TAG point (Fig. 1A).

Crater ejecta, especially in the bulk excavation phase, typically leaves the surface in a ∼45° cone from the expanding crater cavity, and late-stage ejecta can leave at lower angles relative to local horizontal (Cintala et al. 1999; Anderson et al. 2003). The continuous height increase from the crater center (near the TAG point) to Mt. Doom suggests that late-stage ejecta from the Hokioi-forming impact was blocked from leaving the crater in the southeast direction. We expect that this material fell back into the crater and contributed to the asymmetric mound shape extending from the rim into the center. Consistent with this hypothesis, mapping of Hokioi crater before TAG (Barnouin et al. 2022) did not identify any ejecta beyond the crater rim in the direction of Mt. Doom.

In this paper, we describe the vertical position of TAGSAM relative to Bennu in two ways. When describing the vertical distance into the surface that TAGSAM traveled from the TAG point, we use “depth”. When describing TAGSAM’s vertical location relative to the lowest location in the pre-TAG surface (white star in Fig. 1B) — which is the reference zero height in the local DTM — we use “height”.

### The Sequence of Energy Deposition into the Surface

**Fig. 2:**
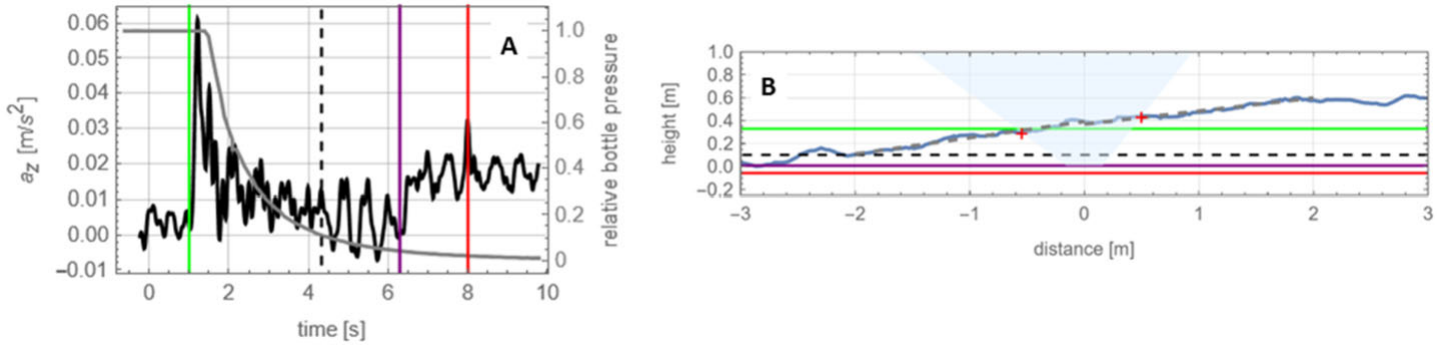
Sequence of events in time and in depth. **(A)** The spacecraft vertical acceleration (black line, left vertical axis) and relative sample gas-bottle pressure (gray line, right vertical axis), as a function of time relative to first contact. The green vertical line is when the TAGSAM gas is released, the purple vertical line is when the backaway thrusters start, and the red vertical line is when the spacecraft vertical speed reaches zero, marking the greatest depth in the subsurface. The black, dashed vertical line marks a transition in the acceleration after the TAGSAM bottle is essentially expended. **(B)** A profile (blue line) across the y = 0 DTM (see Fig. 1B). The horizontal axis is distance from the TAG point, and the vertical axis is height. The colored horizontal lines are the depths of the events marked by the corresponding vertical lines in (A). Gray dashed lines are linear fits to the profile from −2 to 0 m and from 0 to +2 m. A light blue frustum is a 45° cone, starting at the purple line, rotated so that the frustum has 45° exit relative to each gray line. The frustum represents the geometry of TAGSAM ejecta at the time the thrusters turn on. Red crosses are the intersection between the frustum and the surface profile

The spacecraft’s deposition of energy and momentum into the surface and subsurface during TAG comprised the following key events (Fig. 2A): (i) first contact at T+0 s, (ii) TAGSAM gas release at T+∼1 s, (iii) initiation of backaway maneuver at T+∼6.1 s, and (iv) end of backaway maneuver at T+31.6 seconds (Lauretta et al. 2022). The TAG point had a height of ∼39 cm (Fig. 2B). The first second of contact entailed purely mechanical transfer of momentum and energy from the spacecraft (Walsh et al. 2022; Bierhaus et al. 2021). The firing of the TAGSAM and thrusters injected gas into the regolith. The thrusters initiated when the TAGSAM gas bottle pressure was 2.5% of its starting value (Fig. 2A). Thus, the deposition of energy from TAGSAM and the thrusters is separable, although the surface reaction to these events overlaps. About 1.9 s after the backaway maneuver started, the spacecraft vertical speed reached zero and began to reverse direction away from the asteroid. TAGSAM was ∼3.6 m above the pre-contact surface when the spacecraft thrusters turned off.

### The Initial Crater Formed by TAGSAM

The injection of TAGSAM gas mobilized a significant amount of material (Bierhaus et al. 2021; Lauretta et al. 2022). By design, the TAGSAM gas was essentially expended before the backaway maneuver began. To constrain the size of the TAGSAM-generated crater at the moment before the spacecraft thrusters turned on, we apply two methods, each of which uses established ejecta behavior (Housen and Holsapple 2011) and is grounded in TAGSAM test data (Bierhaus et al. 2018, 2021). The first method considers the relationship between crater growth and visible ejecta. The second method considers the depth of TAGSAM and the corresponding geometry of material motion from the subsurface to the surface. The two analyses are independent and complementary: one considers the growth of the crater rim and the corresponding evolution of ejecta visible to the spacecraft cameras, and the other considers processes within the subsurface that drive surface-ejection geometry. As described in more detail later, the super-position of TAGSAM and the thrusters resulted in a deeper central region that transitions to shallower, then irregular, erosion. In the context of TAGSAM-only excavation we use the term crater, though describe the combined effects as a TAG erosion region.

#### Navigation and Sample Camera Views of the TAG Event

The first method to evaluate the TAGSAM crater size uses images obtained by the Navigation Camera (NavCam, Bos et al. 2018). NavCam images were acquired at about one per second throughout the TAG event, including descent, TAGSAM gas release, and the backaway thruster firing (Supplemental Movie S1). As a result the images capture the initial mobilization of Bennu material from TAGSAM (Lauretta et al. 2022), as well as the evolution of ejecta within the camera FOV during backaway. When the spacecraft is close to the surface (∼a few m) the FOV is small and captures a limited perspective of the ejecta; however, as the spacecraft range increases the camera FOV expands to include a broad view of ejecta around the TAG location. Because of the camera orientation on the spacecraft, the NavCam view is offset to the ∼north-east of the nadir direction.

**Fig. 3:**
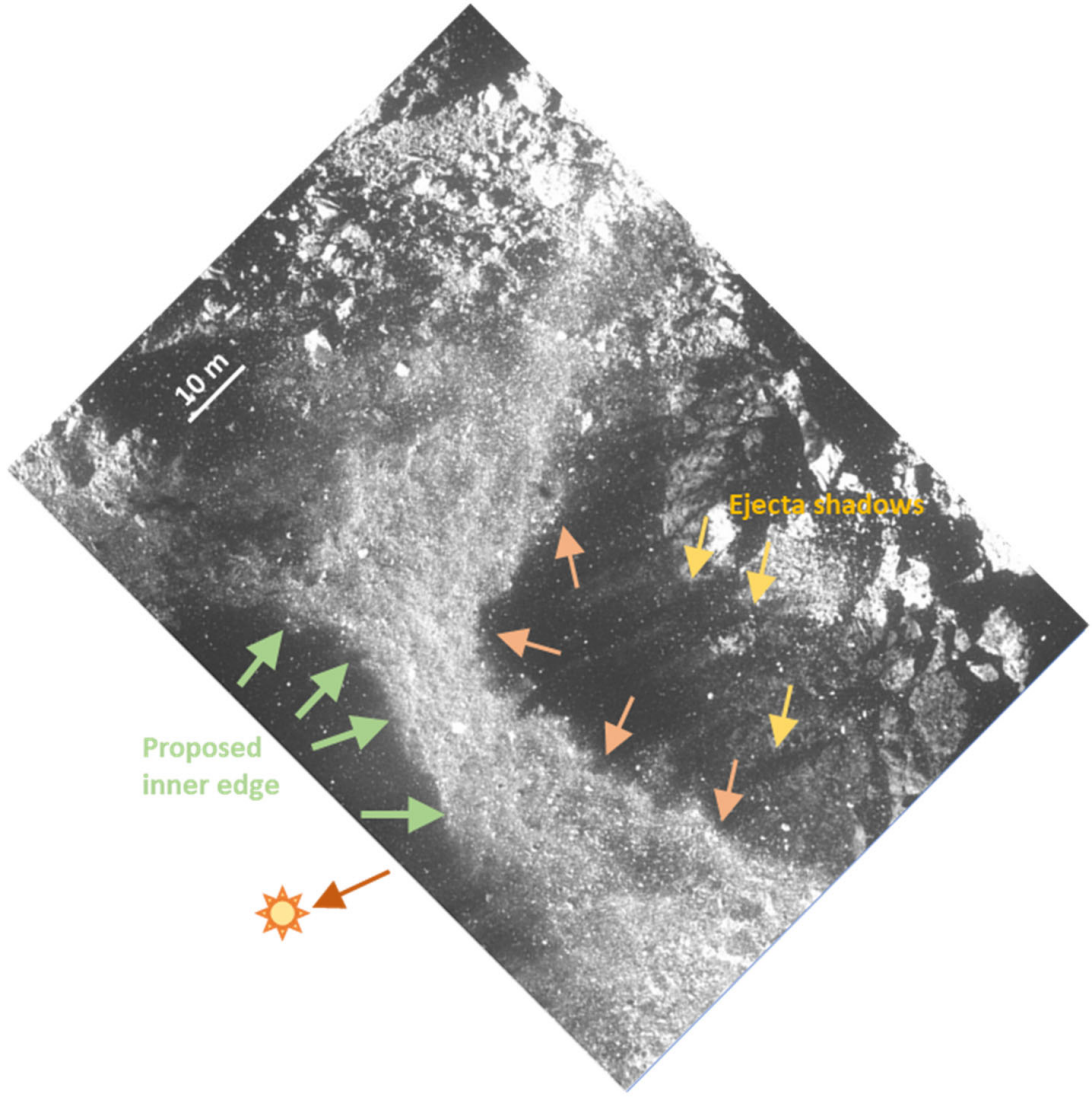
A view of Bennu during spacecraft backaway after TAG. The NavCam image (20201020T215107S676) is ∼77 s after first contact, and the spacecraft was ∼149 m from Bennu. North is approximately up, and the dark orange arrow indicates the approximate Sun direction. This image provides a context view for the TAGSAM ejecta relative to the underlying surface and illustrates the solar illumination geometry and corresponding shadowing of the ejecta. The TAG location is off the image, near the word “edge”, thus the entire scene is eastward/north-eastward of the TAG point. The bright band of particulates running through the image is TAGSAM ejecta above the pre-existing surface. The 10 m scale bar is for features on the ground and is not applicable to the mobilized material. The light rose arrows indicate eastern edge of the ejecta, modified by the spacecraft thruster (see Sect. 3.4 for more details). The yellow arrows point to filamentary shadows cast by the ejecta on Bennu’s surface. The green arrows point to a discrete boundary on the other side of the ejecta, which is closest to the TAG point – thus the “inner edge”. In addition, there are no adjacent filamentary shadows on the ejecta. The edge follows an elliptical shape that is consistent with the approximate elliptical hill of the pre-TAG surface. The green arrows also indicate the approximate direction of motion for the ejecta

**Fig. 4:**
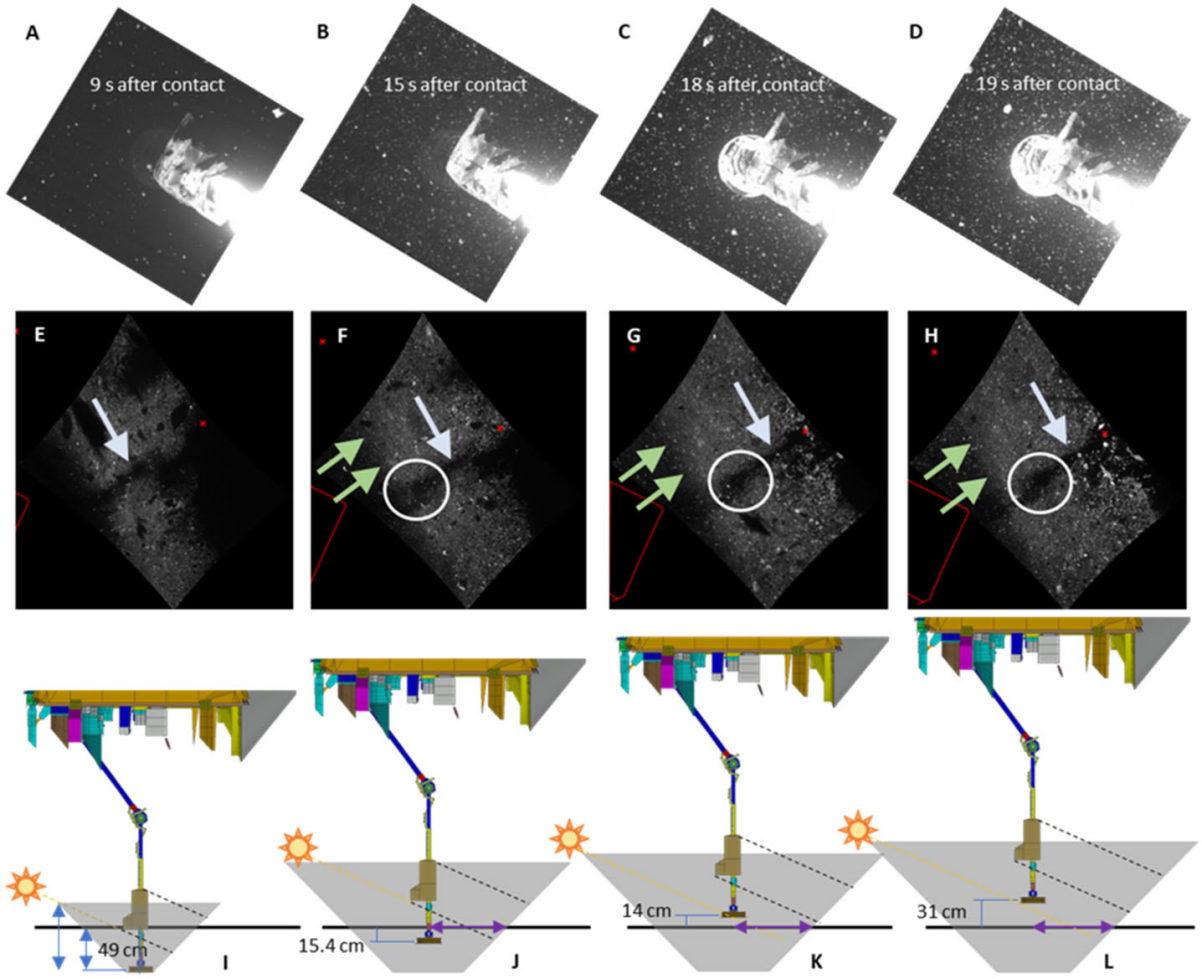
Images and spacecraft geometry that constrain the origin of ejecta inner edge. The four columns represent four different moments from the TAG sequence. Each of the four columns has a Sample Camera (SamCam, Rizk et al. 2018) image (top row), NavCam image (middle row), and schematic of the spacecraft geometry relative to the surface (bottom row). (**A** to **D**) SamCam images 20201020T214958S623, 20201020T215004S709, 20201020T215007S144, and 20201020T215008S361, respectively. These demonstrate that while the TAGSAM was in shadow for part of TAG, much of the arm was not. This fact, plus the emergence of TAGSAM from shadow, provide an important constraint on the height of the opaque ejecta on the western (sunward) side of the TAG location, which was not seen during TAG. In addition, the increase in the number of bright particles from (A) to (D) demonstrates the thruster plumes were recirculating small particles underneath the spacecraft (see Sects. 2.4.3 and 2.4.4). (**E** to **H**) NavCam images 20201020T214958S680, 20201020T215004S680, 20201020T215007S680, and 20201020T215008S680, respectively. The blue arrow points to the shadow of the TAGSAM arm on the ejecta. Inside the white circle is the shadow of the TAGSAM gas-bottle assembly (Bierhaus et al. 2018, and see bottom row of this figure). The green arrows point to the inside edge of the ejecta (the same inner edge noted by green arrows in Fig. 3, though the NavCam images in this figure are much earlier in the backaway sequence). The small red crosses are thruster boresight locations, and the red lines in the lower left of each image indicate the approximate location of the companion SamCam image from the top row. (**I** to **L**) Schematic representation of the geometry for the TAGSAM head, TAGSAM arm, science deck of the OSIRIS-REx spacecraft, the Sun, and the TAGSAM ejecta. The cartoon Sun and yellow dashed line indicate the Sun angle, 66.7° from vertical. The numbers give the TAGSAM height relative to the pre-existing surface, based on propagation of the accelerometer data. The gray frustum is the TAGSAM ejecta. The black dashed lines are the approximate projection of the bottle assembly shadow on the TAGSAM ejecta, consistent with the NavCam images in the row above. The purple line is the approximate radius of the TAGSAM inner crater edge

At the time of TAG the Sun is 66.7° from vertical (Lauretta et al. 2022) to the southwest (Fig. 3). As the camera FOV expands, two shadowed regions appear on either sides of the ejecta (Fig. 3 and movie S1), and a shadow from the TAGSAM arm is visible on the ejecta (Fig. 4). The eastern/north-eastern shadowed region (the one more distant from the TAG point) is cast by ejecta launched in that direction, determined by the Sun location and the ability to correlate features in the ejecta with shadows cast on the surface. The shadowed region on the other side of the ejecta – towards the TAG point – has distinct characteristics (Fig. 3): (i) there is a relatively abrupt boundary between the ejecta and shadow that is morphologically distinct from the filamentary nature of the shadow on the eastern side, and (ii) the shadow inner-edge geometry is approximately elliptical with an orientation consistent with elliptical geometry of the raised region surrounding the TAG point (Fig. 1).

We use the spacecraft location, derived from propagation of accelerometer data, in conjunction with Sample Camera (SamCam, Rizk et al. 2018) images and NavCam images, to evaluate the geometry of the TAGSAM relative to the TAGSAM ejecta and the pre-existing surface (Fig. 4). While the TAGSAM is in shadow after sample-gas release and initial backaway, the TAGSAM arm is never in shadow; in addition, SamCam images tightly constrain the emergence of TAGSAM from shadow (Fig. 4 A-D). The NavCam viewing direction and solar illumination direction were fortuitously aligned so that the TAGSAM arm cast a shadow on the TAGSAM ejecta visible in the NavCam images (Fig. 4 E-F). The combined observations, in time, of TAGSAM and the TAGSAM arm shadow provide constraints on the nature of the inner-edge of the ejecta (Fig. 4 I-L).

The absence of filamentary shadows on TAGSAM ejecta, in combination (Fig. 3 and Fig. 4) with (1) the Sun angle, (2) the spacecraft/TAGSAM arm orientation and size, (3) the evolution of the shadowed regions on either side of the ejecta, (4) the TAGSAM arm shadow on the ejecta, and (5) the time evolution of these features in the SamCam and NavCam images enable us to conclude that the western edge of the TAGSAM ejecta seen in the NavCam images is not caused by shadowing from TAGSAM ejecta to the west of the TAG point. In addition, later we show the dynamic pressure associated with the location of the inner edge of the ejected material is more than one order of magnitude smaller than the minimum dynamic pressure from the thrusters that redirects the bulk of TAGSAM ejecta (see Sect. 3.5 for more details). We therefore conclude that this inner edge to the ejecta corresponds with the growth of the TAGSAM crater, and was not caused by the thrusters.

#### TAGSAM Crater Size Derived from Images of Ejecta Motion

**Fig. 5:**
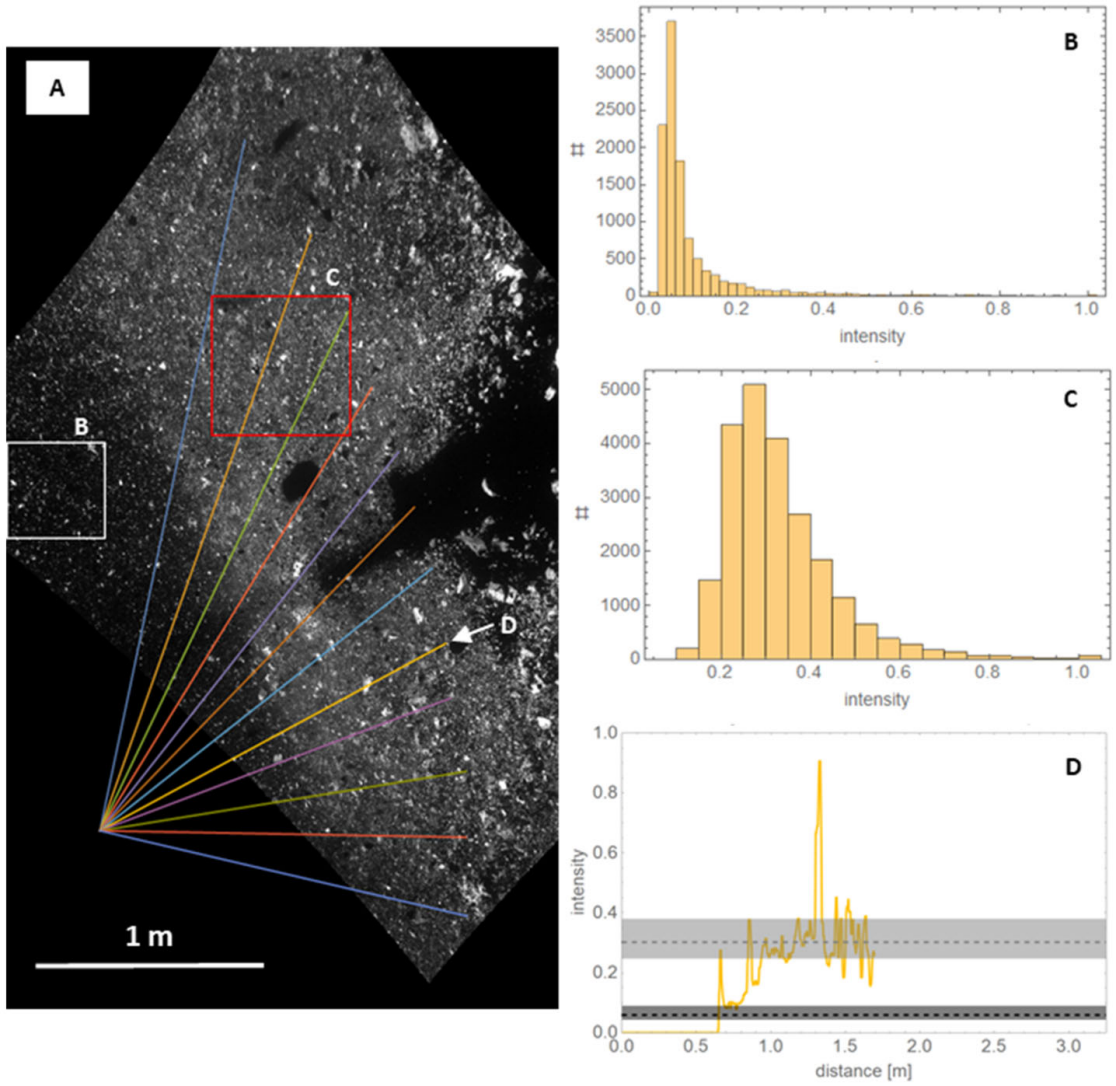
Tracking motion of the inner edge of TAGSAM ejecta. **(A)** NavCam image 20201020T215012S680, projected with north up. The white box outlines a region interior to the TAGSAM ejecta. The red box outlines a region on the TAGSAM ejecta. The lines of various colors radiate from the TAG point, and cross onto the TAGSAM ejecta at different azimuths. **(B)** The distribution of image-normalized brightness values within the white box shown in (A). **(C)** The distribution of image-normalized brightness values within the red box shown in (A). **(D)** The intensity profile along the yellow profile (fifth profile from the bottom). The black dashed line is the median pixel value within the white box, and the dark gray band is the 25–75% range of pixel values within the white box. The gray dashed line is the median pixel value within the red box, and the gray band is the 25–75% pixel value within the red box. We define the transition of the brightness into the lower bound of the gray box as the location of the inner edge of the TAGSAM ejecta

We use observations of the inner edge of the TAGSAM-driven ejecta to estimate the pre-thruster TAGSAM crater size. Critically, the NavCam images contain the inner edge of the TAGSAM ejecta, i.e., the last material ejected from the TAGSAM-caused crater (see Sect. 2.3.1). We projected the NavCam images to a coordinate frame centered on the TAG point with north up (Fig. 5) using a ray tracer and camera model implemented in ISIS3 (DellaGiustina et al. 2018; Keszthelyi et al. 2014). The distribution of normalized pixel brightness values quantify the expected brightnesses for pixels within and outside the TAGSAM ejecta (Fig. 5 B and C). For images that span ∼13 s after the first time the inner edge is visible in the NavCam images, we made multiple brightness profiles that start at the TAG location and extend radially outward, intersecting the inner-edge at multiple locations (Fig. 5A). Using brightness values along the profile to measure when the profile transitions from the dark crater interior to the brighter ejecta, we make distance measurements of the ejecta edge (Fig. 5D).

**Fig. 6:**
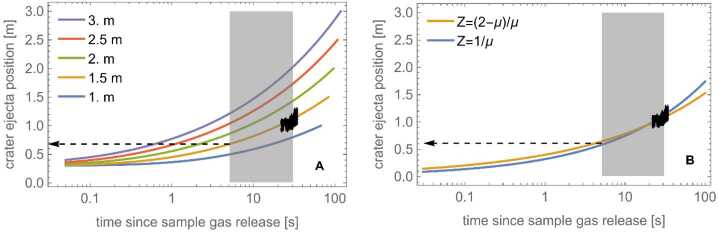
The time-evolution of the TAGSAM inner ejecta edge. **(A)** The multi-colored curves are the growth in time of a hypothetical TAGSAM-caused crater for different final-crater radii (see Sect. 2.3.4). The black points are the observed location, with error bars, in NavCam images of the ejecta inner edge over a span of 13 s. The gray rectangle is the duration of the backaway thruster maneuver. The black dashed arrow points to the crater radius, about 0.68 m, at the time the thrusters start for the radius growth curve that aligns with the black points. The observations fall on the curve for a 1.5 m radius final crater. **(B)** The same comparison for the Z-model, with two different definitions of Z (see Sect. 2.3.4)

The FOV of the NavCam images acquired prior to thruster initiation does not have the proper orientation to view the initial onset of the inner TAGSAM edge. Therefore, we use NavCam images from later in the sequence whose FOV does include the ejecta edge; the resulting data set is more than 60 measurements of the edge location, distributed over 13 s, a time resolution of ∼1 image per second, with a median of five profile measurements per image. From the observed expansion of the inner edge, we extrapolate back to the time at which the thrusters started; the data correspond to a TAG crater radius of 0.68 m when the thrusters first turn on (Fig. 6).

#### TAGSAM Crater Size Derived from Subsurface-to-Surface Geometry

The classical angle for crater ejecta during bulk excavation is 45° (Cintala et al. 1999; Anderson et al. 2003). Ground tests observed TAGSAM-driven ejecta using high-speed video (Bierhaus et al. 2021) and found an ejection angle ∼45°. The TAGSAM head was not obscured by regolith throughout the entirety of the sampling event at Bennu (Bierhaus et al. 2023), supporting the expectation that ejecta from Bennu behaved similarly. Thus, we define a TAGSAM crater geometry during the sampling gas release using an inverted 45° cone frustum, where the narrow base is the edge of the TAGSAM at depth for a given time, and the wider top is the intersection of a 45° angle from that depth to the surface.

To determine the depth of TAGSAM just prior to spacecraft-thruster activation, and therefore the surface exit radius of TAGSAM ejecta, we use the spacecraft accelerations acquired by the IMU and knowledge of the contact time (Walsh et al. 2022; Lauretta et al. 2022). The vertical speed of the spacecraft at contact was ∼10 cm/s (Lauretta et al. 2022), and designed compliance (Bierhaus et al. 2018) with a surface rock allowed the TAGSAM head to tilt 7° at contact (Walsh et al. 2022). The subsurface depth reached by TAGSAM (after compliance with local tilt) at the time of nitrogen gas fire was ∼6–7 cm. The surface provided measurable but small resistance to the spacecraft (Walsh et al. 2022). The TAGSAM gas caused deceleration of the spacecraft, and in the 5 s between TAGSAM gas release and spacecraft thruster initiation we find (by integrating the IMU accelerometer data) the spacecraft descended an additional ∼32 cm into the surface, reaching a depth of ∼38 cm; this marks the bottom of the inverted frustum at the moment before the thrusters fire. The spacecraft descended an additional 7–11 cm before the thrusters fully halted downward motion. TAGSAM reached ∼45–49 cm maximum depth, depending on how much spacecraft downward motion was accommodated by TAGSAM tilt during first contact. Lauretta et al. (2022) derived a 48.8 cm maximum depth; though they did not account for TAGSAM tilt at first contact, the two maximum-depth estimates are consistent.

To find the surface exit location of the frustum, we project a 45° cone from a 38 cm depth below the contact point and account for the tilt of the surface relative to a horizontal plane (Fig. 2B). To find a local normal around pre-TAG topography, we fit a line from the TAG point to 2 m radial distance, which encompasses the major topographic variation around the TAG point (Fig. 2B). This compensation moves the exit point closer to the TAG point when the surface tilts towards the TAG location and farther away when the surface tilts away from the TAG location.

**Fig. 7:**
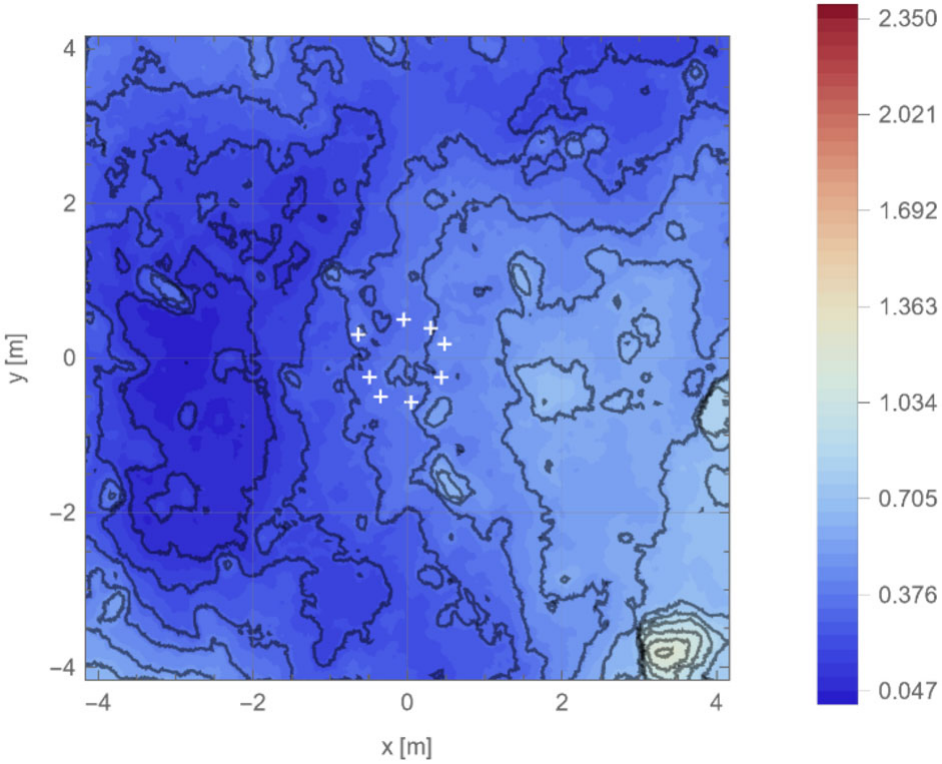
Estimated TAGSAM ejection radii relative to pre-TAG surface just before backaway initiation. This figure is a zoomed version of Fig. 1B, with white crosses marking the surface exit locations of TAGSAM ejecta at multiple locations (using the frustum method from Fig. 2B) the moment before the thrusters start. Contour lines mark 10 cm steps in height

Given TAGSAM’s ∼38 cm depth just before thruster initiation, we calculate ejecta exit locations from this depth along four profiles across the DTM for this moment in time. Each profile provides a pair of exit points (on opposite sides of the TAG point) for a total of eight exit locations (Fig. 7). The median distance of TAGSAM ejecta exit locations from the TAG point is 0.53 m, the minimum is 0.50 m, and the maximum is 0.71 m.

#### Impact-Crater Analogy for TAGSAM Crater Size

We compare the time-evolution of the inner edge with crater-ejecta models that predict the growth of the crater edge as a function of time to test consistency with the locations derived in Sects. 2.3.2 and 2.3.3. The centralized, direct release of TAGSAM gas into surrounding regolith (vs. from a standoff distance, like a thruster) motivates an impact-crater analogy (Bierhaus et al. 2021) to TAGSAM’s diffused gas disruption. Decades of impact experiments and numerical modeling provide the foundation for what are known as crater-scaling relationships, which equate impactor and target properties to a resulting crater, including crater ejecta (e.g., Housen and Holsapple 2011). Analysis of Bennu’s craters and ejecta (Bierhaus et al. 2022; Perry et al. 2022), and the Hayabusa2 SCI experiment on asteroid Ryugu (Arakawa et al. 2020), indicate a strengthless, or nearly strengthless, surface response to cratering in small, rubble-pile asteroids. A strengthless regime corresponds to gravity-dominated evolution of crater growth. Velocity of material ejected in the gravity regime is (Housen and Holsapple 2011):

1 $$ v_{ej} = \sqrt{g R} C_{2} \left ( \frac{x}{R} \right )^{-1/\mu} $$

where

2 $$ C_{2} = C_{1} \left ( \left ( \frac{4\pi}{3} \right )^{1/3} H_{1} \right )^{\frac{-(2+\mu )}{2\mu}} $$

and the variables and values are in Table 1.

**Table 1:** Symbols, units, and values used for TAGSAM’s impact-crater analogue

Symbol	Value	Unit	Description
Target properties			
g	5.42×10^−5^	m/s^2^	surface acceleration, a combination of surface gravity and rotation [26]
$\rho _{t}$	1200	kg/m^3^	Bennu bulk density (Scheeres et al. 2019)
Impactor (TAGSAM) properties			
$\rho _{i}$	0.036	kg/m^3^	Nitrogen gas density
$r_{i}$	0.15	m	TAGSAM radius
$m_{i}$	0.061	kg	Sample gas mass
$v_{i}$	300-400	m/s	TAGSAM gas speed (Bierhaus et al. 2021)
*E*	11	kJ	TAGSAM gas potential energy
Housen and Holsapple crater scaling-relationships parameters			
*μ*	0.41	-	empirical constant, value for sand (Housen and Holsapple 2011)
*ν*	0.4	-	empirical constant, multiple materials (Housen and Holsapple 2011)
H_1_	0.59	-	empirical constant, value for sand (Housen and Holsapple 2011)
C_1_	0.55	-	empirical constant, value for sand mixture (Housen and Holsapple 2011)
R	various	m	transient crater radius
x	various	m	Position of crater ejecta

Companion expressions from the crater scaling laws provide estimates of the crater size (Housen and Holsapple 2011), given certain parameters describing the impactor and the surface:

3 $$ R= \left ( \frac{\rho _{t}}{m_{i}} \right )^{-\frac{1}{3}} H_{1} \left ( \frac{\rho _{t}}{\rho _{i}} \right )^{\frac{2+\mu -6\nu}{3* \left ( 2+\mu \right )}} \left ( \frac{g a}{v_{i}^{2}} \right )^{\frac{-\mu}{2+\mu}} $$

**Fig. 8:**
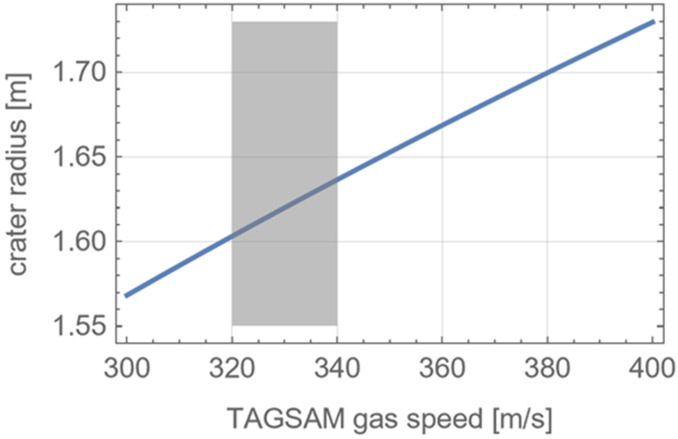
Estimated TAGSAM-only crater size using crater-scaling relationships. The estimated TAGSAM-only crater radius as a function of the effective sample-gas speed. The gray band is predicted range of TAGSAM gas speeds (Bierhaus et al. 2021)

The functional equivalent to the impactor speed is the TAGSAM gas speed; using values expected at TAG (Bierhaus et al. 2021) the final crater radius is ∼1.6–1.65 m (Fig. 8), which is consistent with the 1.5 m radius derived from observing the crater-edge growth (Fig. 6).

We consider another aspect of the impact-crater analogy for TAGSAM: the relationship between the depth of energy deposition and the crater diameter. Melosh (1989) includes a plot demonstrating that the scaled crater diameter changes as a function of the scaled depth of burst $d_{\mathrm{b}}$, where scaled is a length (either diameter or depth) normalized by the cube root of the impact energy, and $d_{\mathrm{b}}$ is approximately the depth at which one can consider the projectile deposits its energy. The scaled diameter reaches a maximum at $d_{\mathrm{b}} \sim 0.003$ – 0.004; shallower cases transmit less kinetic energy into mobilizing surrounding regolith, while deeper cases are less able to mobilize the increased mass of overlying material. Melosh notes that for impacts $d_{\mathrm{b}}$ is nearly zero, i.e. much less than the values that maximize scaled crater diameter. TAGSAM was in Bennu’s subsurface for the entirety of the sample gas release, although the depth was not constant. The TAGSAM depth at 50% of initial pressure was ∼16 cm, and the TAGSAM depth at 10% original bottle pressure (90% spent) was ∼26 cm. Using those depths with the TAGSAM potential energy and $d_{\mathrm{b}}$ = depth/energy^1/3^, the TAGSAM $d_{\mathrm{b}}$ is of order 0.007 – 0.012. This value is deeper than the scaled depths for impacts, however, because scaled diameter decreases beyond $d_{\mathrm{b}} \sim 0.004$, the scaled diameter for the range of TAGSAM $d_{\mathrm{b}}$ is similar to that of impact craters.

To compare with the Z-model (e.g., Maxwell 1977) of crater excavation, we follow Kurosawa and Takada (2019), who describe the crater radius growth as $R$_cr_ $\propto t^{2/(Z+3)}$, where $t$ is time and $Z$ is a parameter related to the velocity decay with time (Maxwell 1977). Kurosawa and Takada (2019) note that Z can be alternately defined as $1/\mu $ or $(2-\mu )/\mu $. We consider both definitions, and introduce a scaling factor to R to align the growth rate with the data. The resulting combinations are $R_{\mathrm{cr}} \sim 0.32 t^{2/(Z+3)}$ for $\mathrm{Z} = 1/\mu = 2.4$, and $R_{\mathrm{cr}} \sim 0.40 t^{2/(Z+3)}$ for $\mathrm{Z} = (2-\mu )/ \mu = 3.8$ (Fig. 6). The corresponding crater radius at the time the thrusters activate is ∼0.61 m, slightly smaller than the 0.68 m derived via crater scaling laws, and within the 0.5–0.71 m predicted for the 45° ejection angle of material mobilized by TAGSAM from the subsurface. In addition, the result is consistent with Kurosawa and Takada (2019), who find the Z-model regularly predicts smaller crater sizes than the crater scaling laws.

#### Collation of Derived TAGSAM Crater Sizes

The previous subsections provide multiple independent methods to estimate the TAGSAM crater size when the backaway maneuver started, as well as the time evolution of the crater growth.

The derived radius of the TAGSAM crater just before thruster initiation:

- Tracking the growth of the inner edge of the ejecta, Sect. 2.3.2: the derived TAGSAM crater radius is 0.68 m.
- Estimating crater size from TAGSAM and ejecta geometry, Sect. 2.3.3: the derived TAGSAM crater radius is 0.50 to 0.71 m

Thus, we conclude that just before the thrusters turned on, TAGSAM was ∼38 cm below the contact point; at the surface, the TAGSAM crater was 0.5–0.7 m in radius and still expanding; and the varying surface heights neighboring the TAG location (Fig. 1) mean that the volume of material available for ejection varied azimuthally. These are the initial conditions of the environment when the spacecraft thrusters fired. We revisit final-crater sizes in Sect. 3.3 and Sect. 4.

### The Interaction of Spacecraft Thrusters with Bennu’s Surface

#### Brief Background on Physical Processes

The Apollo astronauts observed regolith motion caused by the lunar lander thrusters (e.g. Metzger et al. 2011; Hutton 1968; Land and Scholl 1969). Subsequently, all lander thrusters used at the Moon and Mars have been observed to disturb regolith (Mehta et al. 2013; Clegg-Watkins et al. 2016; Lorenz 2016). The physical processes involved are complex because gas-plus-regolith motion is a two-phase flow. Recent advances in analytical formulations and testing (Metzger et al. 2009a,b, 2012; LaMarche 2013) provide clarifications to the driving dynamics. Four thruster-driven processes cause regolith mobility (Metzger et al. 2009a): viscous erosion, bearing-capacity failure, diffused-gas eruption, and diffusion-driven flow. Viscous erosion removes the top layer of regolith via shear stress. Bearing-capacity failure erodes material to form a cup-like depression beneath the stagnation region of the impinging plume. Diffused-gas disruption involves the migration of gas through pores, and in the case that the gas pathway reaches the free boundary of the regolith surface, the gas will “erupt”, carrying regolith away from the surface due to gas drag. In diffusion-driven flow (Metzger et al. 2021), a constantly resupplied stagnation pressure from the thrusters pushes gas through the regolith, causing the regolith to shear and mobilize. Both diffusion-driven flow and bearing-capacity failure can excavate in bulk, i.e., in the case of a single thruster the exhaust forms a thruster-driven crater (Metzger et al. 2012); the former process moves soil tangential to the crater surface, whereas the later excavates by pushing perpendicular to the surface.

Past work suggested he asymptotic depth of a thruster-caused crater can be characterized by an erosion parameter (Rajaratnam and Beltaos 1977; Metzger et al. 2009a,b, 2012; LaMarche 2013), which is based on the densimetric Froude number:

4 $$ F_{0} = \frac{v}{\sqrt{\frac{g D \Delta \rho}{\rho}}} $$

Where $F_{0}$ is the densimetric Froude number, $v$ is the jet velocity, $g$ is surface gravity, $D$ is a representative diameter of the particle population, $\rho $ is the gas (or fluid, depending on the circumstances) density, and $\Delta \rho $ is the difference between the gas and the particle density. Related work by Rajaratnam and colleagues (e.g. Rajaratnam 1981; Mazurek and Rajaratnam 2005) define an erosion parameter that includes simple factors related to thruster-surface geometry, and the thruster itself:

5 $$ E= \frac{F_{0}}{\sqrt{\frac{H}{2 b}}} $$

Where $H$ is the slant range between the nozzle exit and the surface, and $b$ is the nozzle radius (2$b$ is the nozzle diameter). In these formulations, if all other parameters are held fixed but gravity changes, the regolith mobilization will scale as $\sim 1/ \sqrt{g}$. Comparing Bennu – which has a mean $g_{\mathrm{B}}$ of $5.4{\times}10^{-5}\text{ m}/\mathrm{s}^{2}$ (Daly et al. 2020a) – with the Moon, $g_{\mathrm{M}}$ = 1.62 m/s^2^, the Bennu-to-Moon ratio is $\sqrt{1.62}/\sqrt{5.4\times 10^{-5}}= 173$, i.e. the same thruster will excavate almost 200 times deeper at Bennu than at the Moon.

However, as summarized by Metzger (2024a), later studies did not converge on whether the erosion scales with momentum or kinetic energy. In addition, cohesion can matter for granular systems. Based on evaluation of experimental data and thruster plume characteristics in a vacuum, Metzger (2024a) derived a revised formulation for a mass erosion rate:

6 $$ \dot{m} = \rho _{b} \frac{\varepsilon \left ( \frac{1}{12} \rho _{0} v_{0}^{2} \overline{v_{T}} - E_{th} \right )}{\rho _{b} g \left \langle D \right \rangle +\alpha} $$

Where $\dot{m}$ is the local erosion rate in kg/m^2^/s, $\rho _{\mathrm{b}}$ is the bulk regolith density, $\rho _{0}$ and $v_{0}$ are the local thruster-plume density and velocity, respectively, $\overline{v_{T}}$ is the local thruster-plume molecular thermal velocity, $g$ is gravity, $\langle D \rangle$ is a representative particle diameter, $\alpha $ is the regolith cohesion, $E$_th_ is a threshold value of $E$ at which erosion starts, and $\varepsilon $ is an empirical constant. $E$ is the downward energy flux at a height just above $\langle D \rangle$, defined as $E = \rho _{0} v_{0}^{2} \overline{v_{T}} /12$ (Metzger 2024a). Equation (6) can be considered a ratio between the thruster erosional capability (in the numerator) and the regolith’s resistance to motion (in the denominator). Morris et al. (2015) also suggest that the erosion mass flux is proportional to the gas dynamic flux near the surface. We revisit this formulation in the context of our simulation results, see Sect. 3.6.

#### Modeling Description

We analyzed the interaction between the OSIRIS-REx thruster plumes and Bennu’s surface with DAC (a nested acronym: Direct-Simulation Monte Carlo (DSMC) Analysis Code, LeBeau 1999). DSMC is a method typically used for rarified flows, and directly simulates large numbers of molecules. The DSMC code solves for inter-molecular collisions, surface collisions, internal energy modes, and chemical reactions. This code was developed specifically for understanding gas dynamics in rarified conditions, and has been used to model spacecraft thruster behavior in space (LeBeau and Lumpkin 2001).

Unlike continuum solvers that apply the Navier-Stokes equations with implicit assumptions, DSMC directly models all molecules individually. The modeled molecules are a representative population that capture the distribution of molecules in the real population. The internal energy state, velocity, position, and chemical composition of each molecule in the representative population is stored and tracked throughout the simulation. Each simulated molecule represents a population of real molecules, and simulated molecules are typically generated according to an assumed Maxwellian distribution of real molecules. In the case of volumes with both inflow and outflow boundaries, such as this thruster plume analysis, molecules leave the simulation when they pass through outflow boundaries, and new molecules are constantly generated at the inflow boundary surfaces (i.e., the thrusters). The simulation proceeds in time-steps, with multiple DSMC code operations within each time step. There is a move step, which propagates molecule motion according to stored velocity data, and a collision assessment step, which evaluates both inter-molecular collisions and surface collisions.

The simulation reaches convergence when the inflow/outflow flux is balanced, and the quantities of interest are unchanged with additional iterations. Macroscopic flow-field properties, such as density and temperature are found from the solution data directly by sampling and integrating over flow-field volumes within the simulation.

Derived quantities such as pressures are computed from the macroscopic properties. Shear, pressure, and heat flux are found from the surface collision statistical data. Pressure is defined as the fluid force per unit area acting normal to the surface; shear is the force per unit area acting tangential to the surface, and heat flux is the net energy deposited into the surface from the fluid impingement, accounting for the energy of molecules subsequently leaving the surface. The pressure derives from the accumulation of gas on the surface in a stagnation region, or from topography on the surface (i.e., a rock) interfering with the flow of gas. The shear acts parallel to the surface and is the portion of the gas flow moving across the surface element.

#### Simulation Setup

**Fig. 9:**
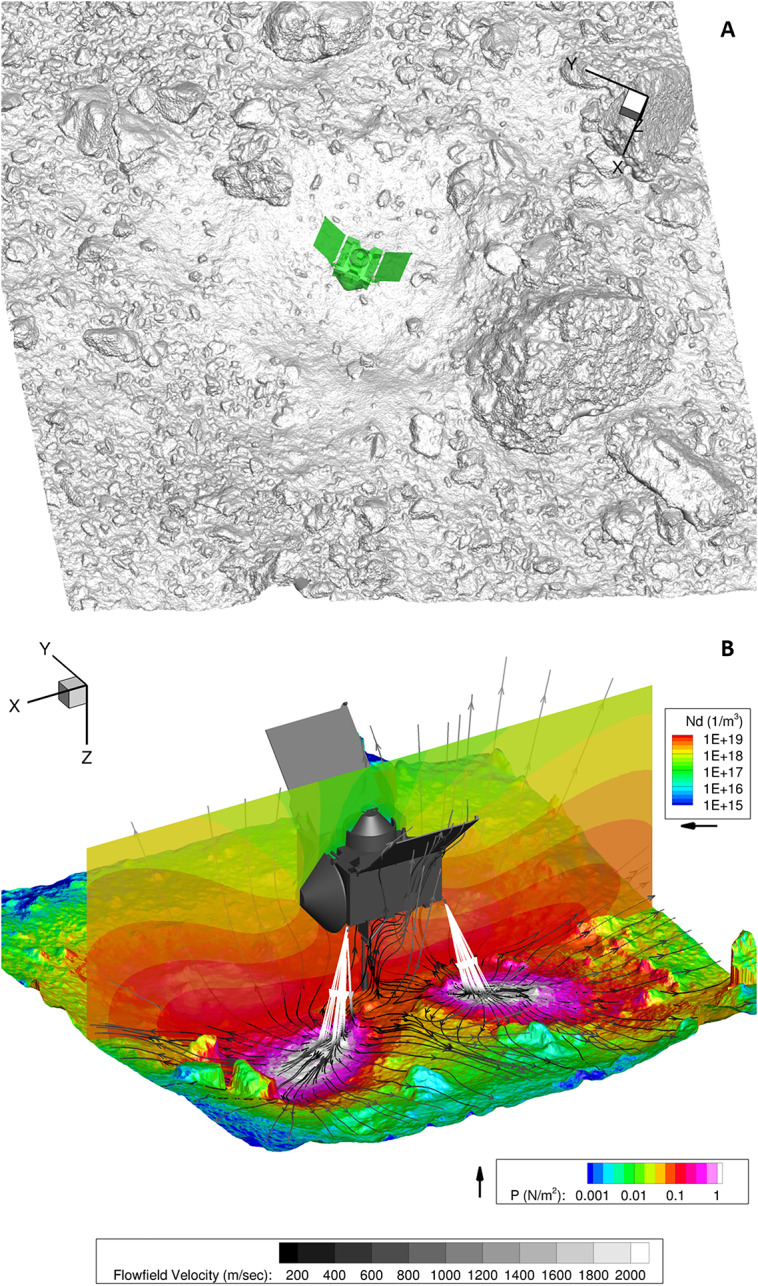
Modeling of spacecraft thruster plumes and related erosional processes. **(A)** The thruster-plume simulation volume included the DTM and the spacecraft. This figure is an accurate representation of the spacecraft location and orientation at TAG. North (+z) and east are approximately up and to the right, respectively. The spacecraft coordinate system at TAG is in the upper right. **(B)** The backaway initiation simulation in 3D. Colors on the surface represent surface pressure (horizontal color legend), colors on the vertical slice indicate the number density of particles in the plume above the surface (vertical color legend), and stream fields and their velocities from the thrusters are plotted in grayscale (horizontal grayscale legend). The spacecraft coordinate system is in the upper left of this panel

**Fig. 10:**
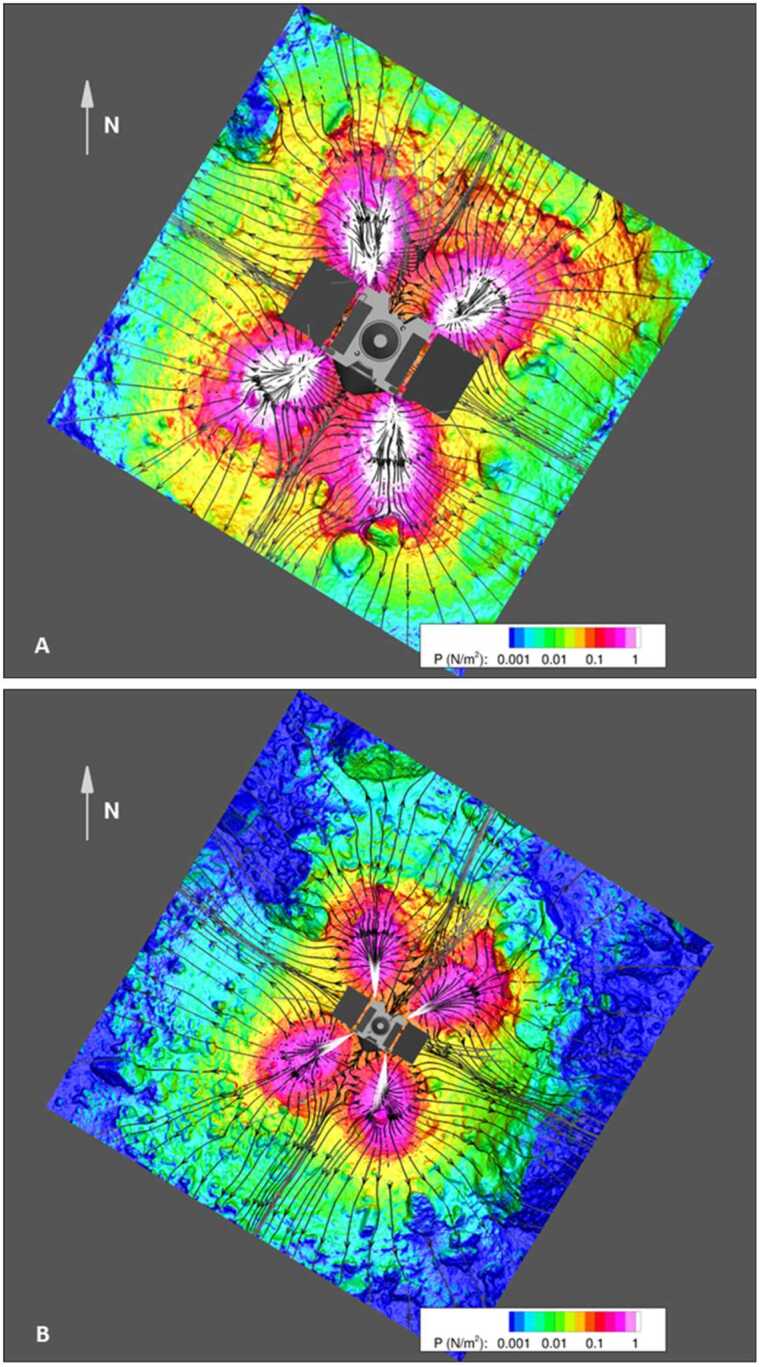
Thruster simulation outcomes for the start and end of the backaway maneuver. **(A)** The distribution of thruster pressure on the surface at backaway initiation. The spatial domain used for this simulation is in Fig. 1B. The black lines are thruster streamlines, indicating the thruster flow direction. **(B)** The distribution of thruster pressure on the surface at the end of the backaway maneuver. The spatial domain used for this simulation is in Fig. 1A

The inlet surfaces for the thrusters used on OSIRIS-REx were developed from continuum CFD (Computational Fluid Dynamics) solutions for the flow-field within the nozzle itself at representative thruster operating conditions. Each of the simulations generated for backaway-start and backaway-end configurations (Fig. 9, Fig. 10) contain approximately four billion molecules, consuming hundreds of gigabytes of memory for the simulation. These gas environments, while enveloping a large domain, are still rarified relative to a traditional planetary atmosphere. Our simulations utilize the pre-TAG site DTM (Lauretta et al. 2022), enabling assessment of pressure build up and flow against the terrain surrounding the spacecraft at TAG.

The pre-TAG surface topography is representative of the majority surface area impinged upon by the thruster plumes. The DTM was adapted slightly to account for computational expectations of the algorithm by projecting a regularly spaced triangular unstructured mesh with a resolution of 5–7 cm onto the source DTM. This process captures the surface topography of the asteroid at the TAG site (Fig. 9A).

In our thruster-plume simulations, the modeled surface fully accommodated the deposition of both momentum and energy, as expected for a porous, non-smooth surface. This treatment does not track gas propagation into the subsurface. The corresponding surface pressure derived for each facet is thus a maximum, though the actual pressure-plus-shear values were likely similar because the (1) the impedance caused by the surface means the majority of the gas did not penetrate the subsurface, and (2) gas flowing into the subsurface would increase the shear on particles, in part or completely counteracting any reduction in pressure.

The surface quantities are influenced by the assumed accommodation coefficient(s), which dictates how energy and momentum will be transferred from the molecules to a surface. In the case of plume impingement on the surface of Bennu, the accommodation coefficient was assumed to be 1.0, which is corresponds to full deposition of gas molecule energy and momentum into the surface. This assumption is appropriate for a porous, non-smooth surface.

Eight, 4.5 N thrusters were used for the backaway maneuver, arranged in four pairs, with one pair for each corner of the approximately rectangular deck shape (Bierhaus et al. 2018) that faced the asteroid at the time of backaway. The thrusters are canted at a 30° angle relative to the spacecraft +z axis, and have an expansion ratio of 74:1. To calculate the effect of spacecraft thrusters on the surface, we implemented a direct simulation Monte Carlo (DSMC) technique that generates individual gas molecules at each thruster exit and propagates the molecules into a 3D volume mesh (Fig. 9). The 3D volume (Fig. 9A) includes the DTM of the TAG site (Lauretta et al. 2022) and the spacecraft (Bierhaus et al. 2018). The simulation generates number density and velocities of particles, and gas pressures at surfaces, enabling derivation of forces acting on a surface within the modeled 3D volume and gas flow-field velocities.

#### Simulation Outcomes

We simulated two cases in the thruster sequence. The first is when the backaway maneuver begins (Fig. 10A), with TAGSAM in the subsurface of the asteroid and the thrusters closest to the surface, capturing the highest pressures and shear effects across the surface (active thrusters at minimum distance). The second is at the end of the backaway maneuver (Fig. 10B), capturing the maximum extent of the thruster imprints across the surface (maximum distance of thruster operation). These two cases bound the range of thruster plume interaction with the volume around the spacecraft and the asteroid surface, capturing both the maximum thruster forces on the surface, and the broadest extent of the thruster-surface interaction.

Where effluent from each of the four pairs of thrusters reaches the surface, we find that they create a “footprint” of higher pressure (Fig. 10 A and B), consistent with other simulations of thruster plumes interacting with planetary surfaces (Plemmons et al. 2008; Metzger et al. 2011; Mehta et al. 2013; Morris et al. 2016). The higher-pressure footprint occurs because the rate of gas deposition onto the surface occurs faster than the gas disperses after reaching the surface (the regolith impedes gas flow more than free space). This causes a buildup of gas that results in a “high pressure” (relatively speaking) region on the surface around the spacecraft (Fig. 9B). This region loses gas as the molecules expand into surrounding lower-pressure regions (including the initially vacuum pore space in the subsurface regolith) but is continuously resupplied while the thrusters remain on. In our simulations we found the lateral scale of the thruster plumes spans the entire simulation area, i.e., all facets have non-zero pressure and shear, though the values vary by several orders of magnitude.

**Fig. 11:**
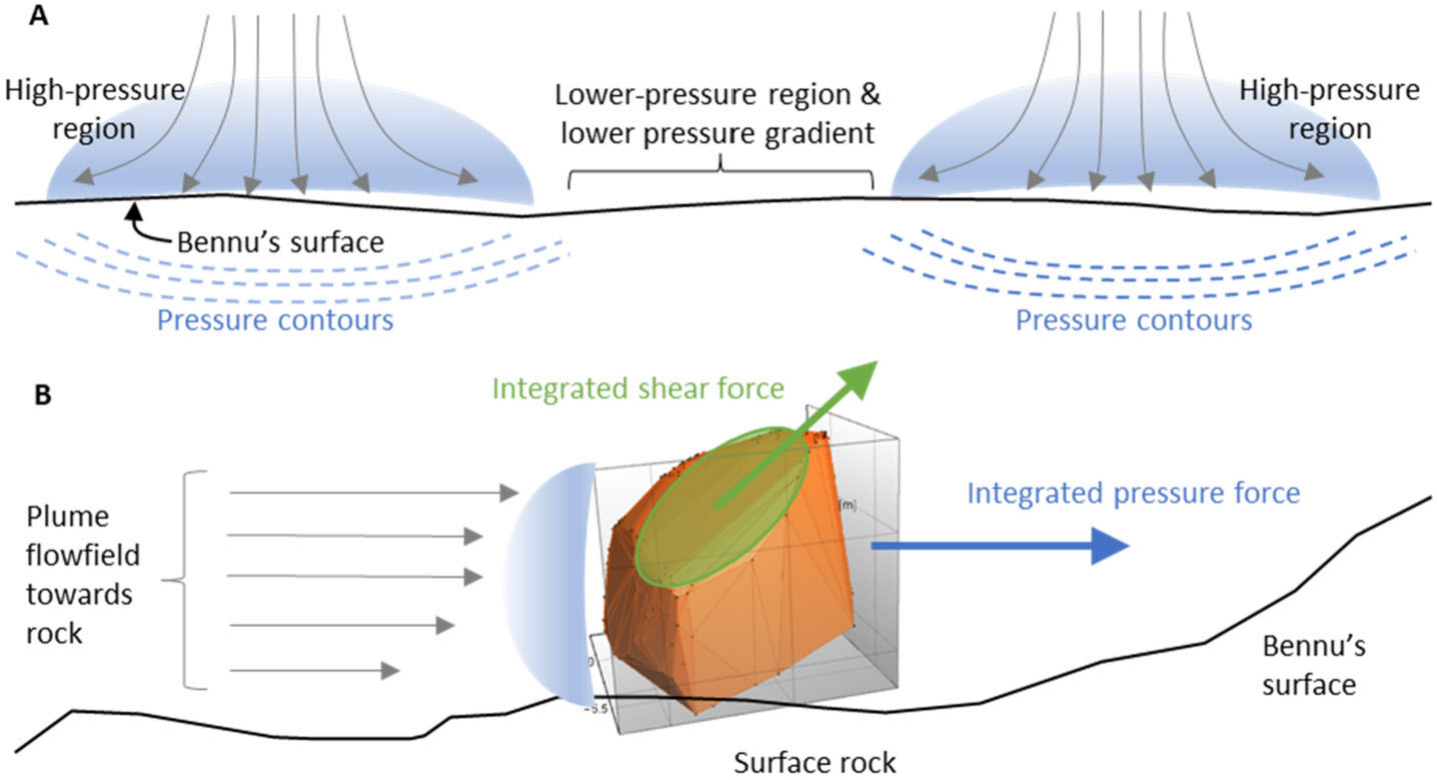
Surface and sub-surface erosional process from the thruster plumes, leveraging the simulation data in Fig. 10. **(A)** Schematic cross section of the near subsurface with two adjacent thruster footprints. The cores of the thruster footprints on the surface create relatively high-pressure regions. In addition to these bubbles expanding across the surface and back into space, these high-pressure regions “pump” gas into the subsurface. The pressure drops across the expansion volume, creating an acceleration gradient. In between the thruster cores are lower-pressure regions with lower acceleration gradients. **(B)** A schematic example of gas flow across the surface interacting with a surface rock. The rock face in the direction of the oncoming gas experiences a high pressure; the leeward side creates a low pressure. Gas flowing across the top and sides of the rock introduces shear. The net acceleration vector is a result of both the pressure and shear

The images acquired during the spacecraft departure from Bennu’s surface do not capture the onset of thruster erosion from the surface; their fields of view when close to the asteroid are obscured by TAGSAM ejecta (e.g. Fig. 4). Thus we cannot empirically derive $\varepsilon $ in Eq. (6). However, we consider the relative importance of different thruster effects summarized in Sect. 2.4.1. Viscous erosion, diffused-gas disruption, and diffusion-driven flow are related to a pressure-driven acceleration created by the pressure gradient inherent in gas expanding into vacuum (Balakrishnan and Bellan 2018). Material will be mobilized where this acceleration is sufficient to overcome the surface gravity and cohesive forces. This will be true for gas expanding into the subsurface and across the surface (Fig. 11). The net effect of thruster-driven erosion is the super-position of (i) bulk-material flow that can form a cavity beneath and near the core of the thruster footprint (either by diffusion-driven flow, bearing-capacity failure, or even diffuse-gas eruption), and (ii) a more surficial erosion that removes regolith primarily by shear (viscous erosion) as the thruster effluent expands across the surface. The second case is analogous to wind-driven particle movement on Earth (Kok et al. 2012), where wind forces must overcome particle cohesion and weight to mobilize material. Fortunately our simulations provide thruster-generated quantities of shear and pressure at the surface, and gas pressure, temperature, and velocity above the surface that enable us to evaluate the thruster effects quantitively (Sect. 3).

### Mapping of Mobilized and Static Particles

**Fig. 12:**
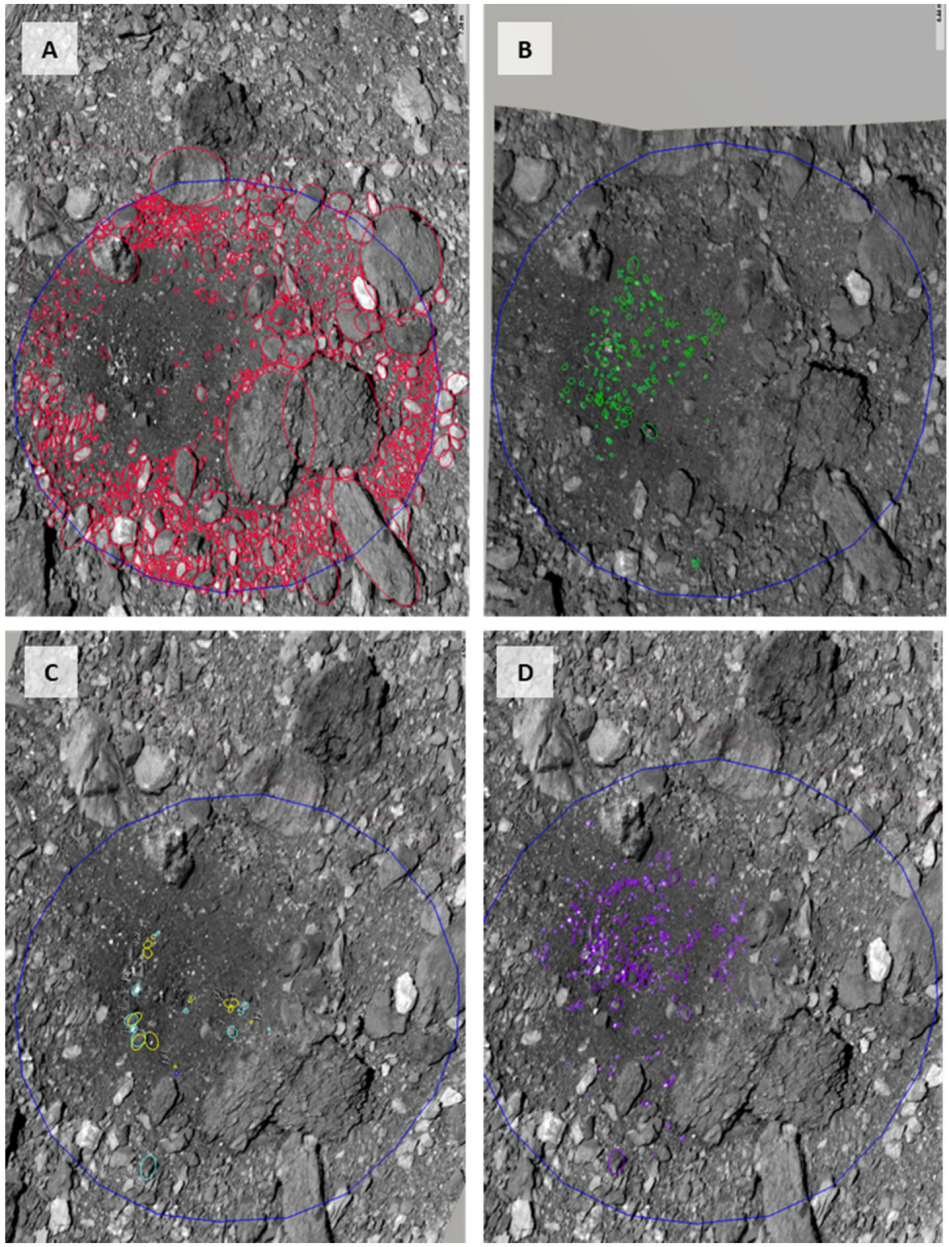
Particle measurements at the TAG site. In all panels, north is approximately up and east is to the right. The blue circle’s diameter in all panels is 38 m, purposefully bigger than Hokioi crater to search for changes beyond the crater rim. We divided the observed particles into the following populations: **(A)** particles that did not move pre- and post-TAG; **(B)** particles that were ejected during TAG; **(C)** particles that shifted during TAG (yellow is the pre-TAG location, teal is the post-TAG location); **(D)** particles that appear in the post-TAG image (and not present in the pre-TAG image)

We used images of the pre- and post-TAG surface (Fig. 12) to evaluate which particles were (i) ejected (removed from the TAG site entirely), (ii) translated (moved but still visible within the TAG region), (iii) static (in the same location pre- and post-TAG), or (iv) present only in the post-TAG surface. The pre-TAG image is ocams20190307t173751s394_pol_iofl2pan.fits and the post-TAG image is ocams20210407t062114s948_pol_iofl2pan.fits. This analysis utilized the Small Body Mapping Tool (SBMT, Ernst et al. 2018) to project the images, with manual identification and measurement of particles. The opacity and pointing of each image were adjusted to align them to each other. This alignment enabled blinking between the images, and the assignment of surface particles to one of the four boulder categories. We defined a 38 m diameter circle centered at the Nightingale TAG point as the area of interest for the detection of boulder categorization and potential movement. This dimension includes all of Hokioi crater and a ∼1 crater radius region beyond the rim.

We measured 1585 unchanged boulders, 193 ejected boulders, 12 shifted boulders, and 258 boulders present in the post-TAG image that are not present in the pre-TAG image (Fig. 12). Some of unshifted boulders (Fig. 12A) appear to be more distinct, suggesting a layer of unresolved fines was removed to sharpen their outlines. The ejected particles (Fig. 12B) follow a roughly rectangular outline that is consistent with the superimposed footprint of all spacecraft thrusters (Fig. 10).

**Fig. 13:**
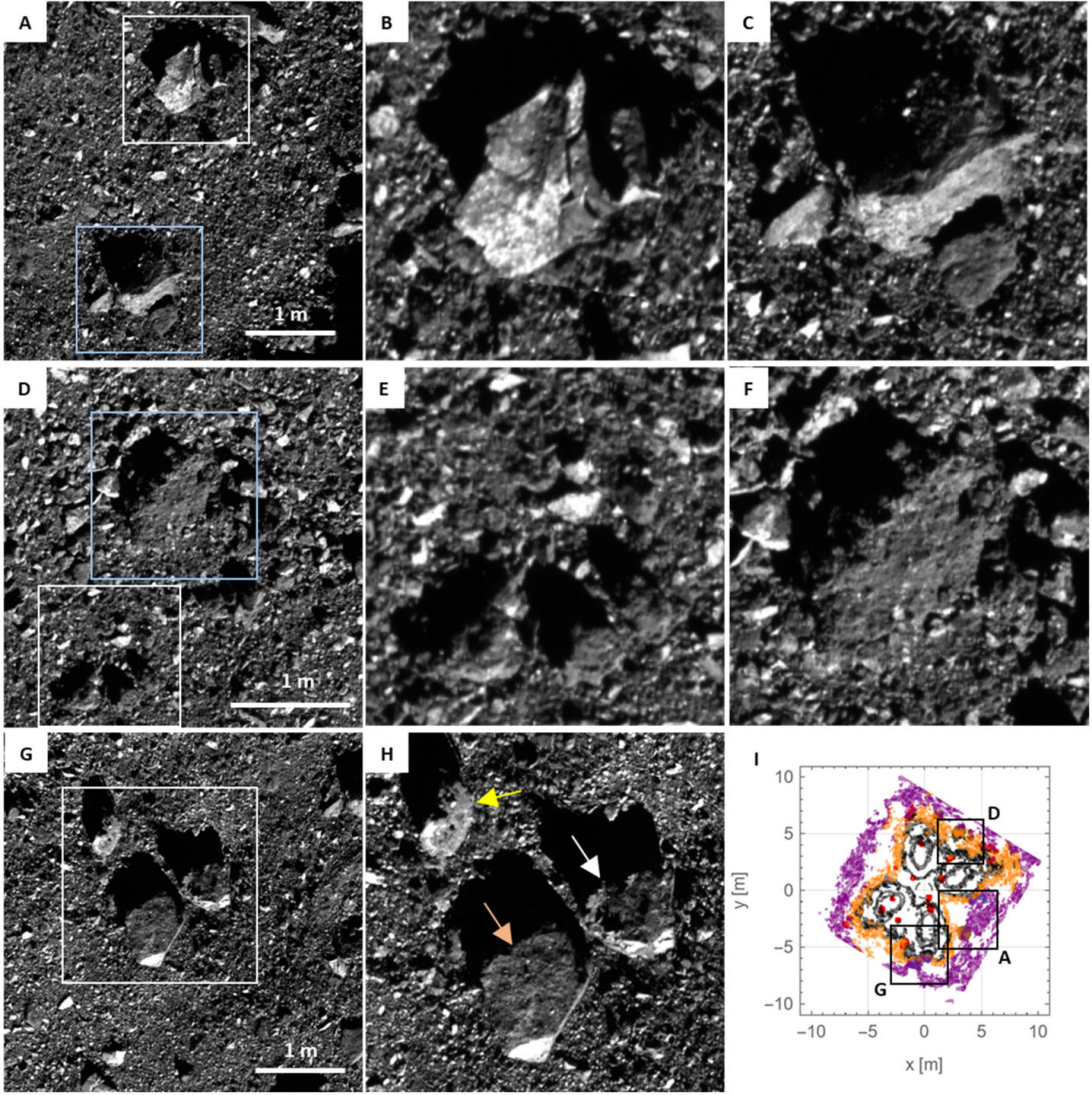
Examples of rocks that were mobilized and static. The mobilized particles (Fig. 12, B and C) appear to rest on the surface, while the static particles (Fig. 12A) are partly buried. **(A)** The white box indicates the zoomed view in **(B)**; the blue box indicates the zoomed view in **(C)**. The thrusters mobilized the rock in (B), but not the rock in (C). **(D)** The white box indicates the zoom in **(E)**, the blue box indicates the zoom in **(F)**. The thrusters mobilized the rocks in (E), but not the rock in (F). **(G)** The white box indicates the zoom in **(H)**. All three rocks arrowed in (H) were mobilized. The white arrow points to the rock that was found ∼12 m from its original location (Lauretta et al. 2022). **(I)** The thruster PPS values from Fig. 10A, along with rocks that were ejected (red), translated (blue), or static (brown). Black squares and companion letters correspond to the views in the panels in the left-hand column of this figure. The white-arrowed rock in (H) was in line with a high PPS contour value, and the contours illustrate this rock partly protected the rock with the light orange arrow in (H)

We used the pre-TAG images to evaluate characteristics of boulders that moved (either ejected or translated) vs. those that remained static. The mobilized particles appear to be resting on the surface, while the static particles appear to have significant volume below the visible surface (Fig. 13).

### Particle Dynamics Observed in NavCam Images

We estimated velocities for particles in the TAGSAM ejecta that are visible in the NavCam backaway images. NavCam collected 133 images at an interval of 1 second; the first image was acquired 46 seconds before initial contact with the surface, and the final image was acquired 87 seconds after contact (∼57 seconds after the thrusters turned off). With a FOV of approximately 40°, this image dataset provides a wealth of information about particle motion and changes to Bennu topography. Prior to the contact phase, NavCam was used to acquire images used by Natural Feature Tracking (NFT; Olds et al. 2022), the automated onboard process used to assess spacecraft position and velocity.

**Fig. 14:**
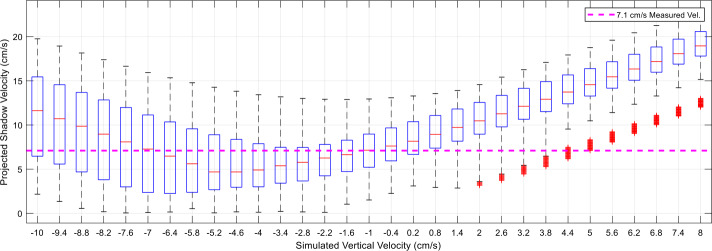
Projected shadow surface velocity results as a function of simulated vertical velocity. In the box and whisker plots above, the central red mark represents the median, the bottom and top edges of the box represent the 25th and 75th percentile respectively, the whiskers extend to the bounds of the data considered inliers, and outliers are plotted individually as a red “+”. Results shown are for the 21 particles that appear connected to the ejecta-plume edge (see Supplemental Information). The dotted pink line represents the 7.1 cm/s measurement of the shadow’s motion over the known rock (Fig. S2)

Our analysis involved measurements from images, knowledge of the spacecraft state, the Sun’s position, and Bennu’s surface (see Supplemental Information) to derive bulk ejecta and individual particle motion. We used the motion of a shadow, cast by TAGSAM ejecta, to derive a projected speed for the bulk ejecta of 7.1 cm/s; this value depends on both the Sun elevation and the ejecta motion. With knowledge of the Sun and spacecraft position, we estimated ∼6.3 cm/s for the vertical component of the bulk speed (Fig. S4). We estimated position and velocity for discrete particles visible on the leading edge of the TAGSAM ejecta (Fig. S5). Use of multiple images and nonlinear least squares minimization provided sufficient data points for a robust solution. As with bulk TAGSAM ejecta, we used shadow lengths to initialize the particle positions, and vertical speeds varying from −10 to 8 cm/s, deliberately larger than the range of plausible values to ensure the minimization did not find a local but incorrect minimum. These combinations resulted in 1456 different vertical trajectories. We estimated the shadow motion cast by the simulated particle trajectories and compared with the observed 7.1 cm/s projected motion described above. Two vertical speeds, −1 cm/s and −7 cm/s, generated the right magnitude of projected motion (i.e., 7.1 cm/s). However, the −7 cm/s trajectories resulted in erroneous shadow directions, whereas the −1 cm/s trajectories were consistent with observed particle motion (Fig. 14).

**Fig. 15:**
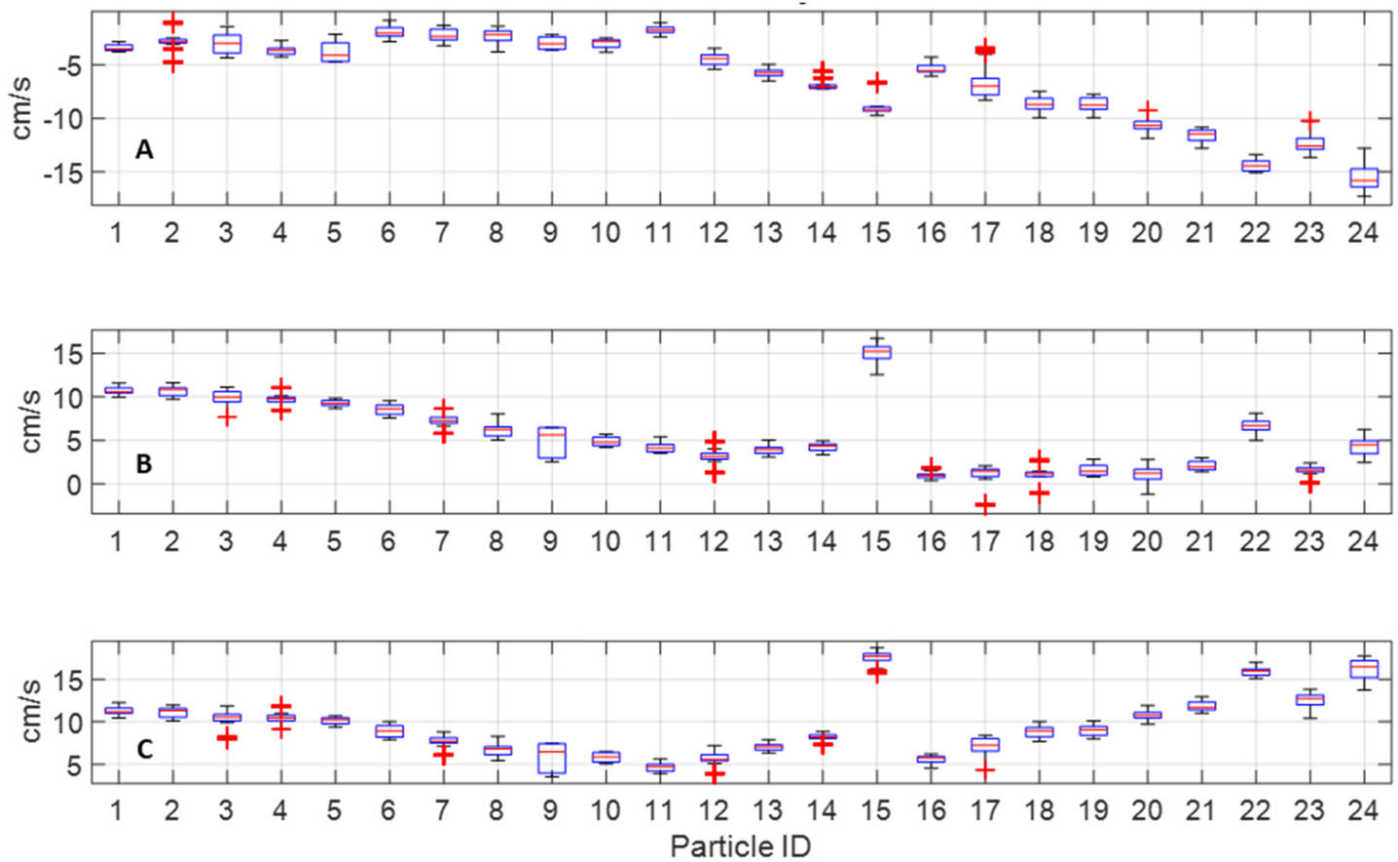
Lateral speed per particle. Results are in a local TAG frame, defined with Z aligned with the surface normal at the TAG location. Particles 15, 23, and 24 are disconnected from the bulk ejecta plume (see Supplemental Information). Particles near the middle of the population (8–14, 16) have lower velocities than particles located towards the “wings” of the plume . **(A)** x-speed, **(B)** y-speed, and **(C)** RSS lateral speed

The data in Fig. 14 combines trajectories with starting heights varying from 0 to 3 m above the surface into each box and whisker data point; analyzing these results further finds that particle vertical velocity of −1 cm/s, with a starting height of 2 m above the surface, results in particle shadows that best match the 7.1 cm/s shadow velocity estimate. Figure 15 shows the corresponding lateral velocities per particle for this simulated vertical trajectory, where particles 15, 23, and 24 are the three particles that move separately from the ejecta plume at higher velocities.

Finally, with estimates of particle positions and velocities, we propagated their states until intersection with Bennu’s surface. Since the images used to measure these velocities were captured 19 seconds after the thrusters turned off, we assumed acceleration other than gravity are zero. For each particle, the lateral velocity used is the average of that particle’s lateral velocity estimate from each image along the best-fit vertical trajectory. We then used that lateral velocity with the best-fit vertical velocity of −1 cm/s, and starting height of 2 m, to propagate each particle’s location to the surface, accounting for Bennu’s gravity and rotation rate. The final particle surface point is the location where the particle height intersects with the surface of the pre-contact DTM, which we address further in Sect. 3.4.

## Results

### Variable Material Properties Within the TAG Area

TAGSAM, and the spacecraft behind it, interacted with an almost 50 cm vertical column (Lauretta et al. 2022). Within this column, we find evidence for multiple transitions in material properties: starting at the surface, there is a ∼10–20 cm layer of easily mobilized material; beneath this layer is a layer that includes finer-grained material; finally, there is a transition to a stronger “basement” coincident with the lowest location in the pre-TAG surface, ∼30–40 cm below the TAG point. In addition, the broad surface area affected by the thruster plumes provides evidence for lateral variations in surface properties. We start with the deepest layer because supporting observations also inform interpretation of the surface layer.

#### Stronger “Basement” Layer

**Fig. 16:**
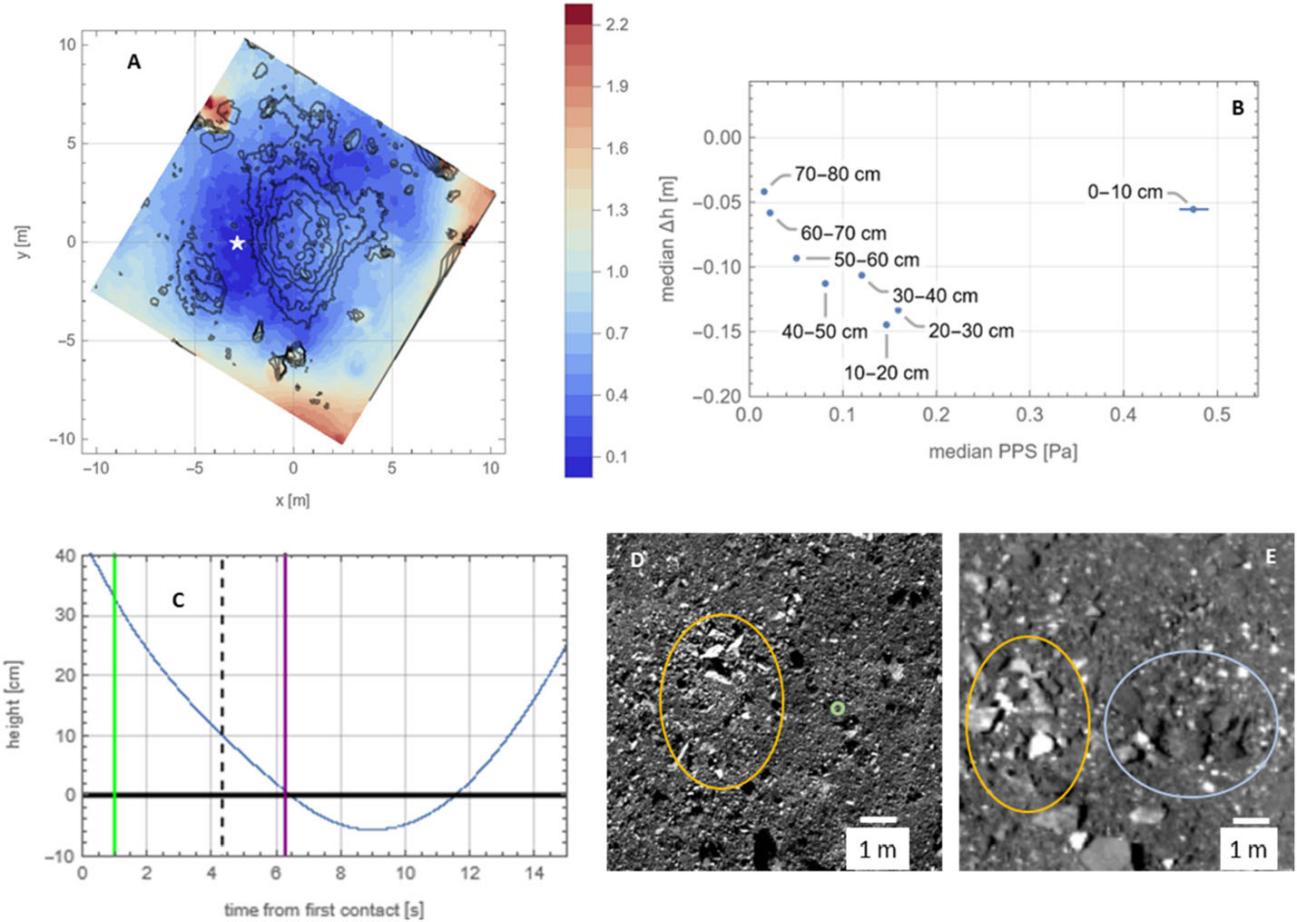
Evidence for a layer of larger rocks in the “basement” of Hokioi crater. **(A)** The pre-TAG surface as rendered in Fig. 1B, except here the black contour lines are for regions that experienced erosion, i.e. they have $\Delta$h < 0, in intervals of −10 cm (the outermost contour of the central region is −10 cm, with successive inward contours decreasing by 10 cm). The white star is the lowest location in the pre-TAG surface. **(B)** The median $\Delta$h (for facets with $\Delta$h < 0) as a function of median PPS, binned according to pre-TAG height. The error bars are the standard error of the median, calculated via bootstrap with replacement. Where error bars are not visible, errors are smaller than the point size. **(C)** The height of TAGSAM during TAG (blue curve), with height defined as in (A). The vertical lines are the same as in Fig. 1C. The acceleration profile in Fig. 1C between the dashed black line and the purple line corresponds to pre-TAG heights < $10\text{ cm}$. **(D)** Pre-TAG context view of the TAG site inside Hokioi crater. The green circle is the approximate TAG point. The yellow oval approximately outlines the lowest regions in the pre-TAG surface, indicated by the white star in (A). **(E)** The post-TAG surface. The yellow oval approximately outlines the same region as in D. The light-blue oval outlines the region around the green circle in (D). Inside both outlines are multiple meter-scale rocks exposed by TAG

Two lines of evidence support a strength increase at greater depths (Fig. 16). The first is the correlation between material removed by the TAG event and the height of the pre-existing surface. The lowest location in the pre-TAG surface is just west of the TAG location (Fig. 1B, Fig. 7, and Fig. 16A). This location, despite residing within a high-pressure region from a thruster footprint, and in contrast to the three other thruster footprints, did not experience any net erosion (Fig. 16A). (We define erosion as $\Delta $h < 0, where $\Delta $h is the pre-TAG height minus the post-TAG height, DTMs from Lauretta et al. 2022). The absence of erosion in this region results in a discontinuity in the $\Delta $h < 0 contours in the western (i.e., x < 0) locations (Fig. 16A); none of the $\Delta $h < 0 contours intersect the lowest portion of the pre-existing surface.

We find that the regions with pre-TAG heights of 0–10 cm responded anomalously to the thruster pressure and shear (pressure-plus-shear, or PPS, which corresponds to accelerations on surface facets; Fig. 16B). Whereas all other heights express a trend of increasing erosion (i.e., increasingly negative $\Delta $h) with increasing median PPS, the 0–10 cm pre-TAG height bin shows the second lowest net removal despite having the largest median PPS value.

The second piece of evidence for a stronger layer is the change in the acceleration profile with depth. The release of the TAGSAM gas caused an initial spike in vertical acceleration that decayed as the gas was expended (Fig. 2A). After the gas release was essentially complete, but before the backaway thrusters were activated, the amplitude of the acceleration increased (see the portion of the profile between the vertical black dashed line and the purple line in Fig. 2A). Because the TAGSAM gas was expended and the backaway thrusters were not yet on, the only source of external force on the spacecraft was Bennu’s subsurface. The height of this acceleration change corresponds to 10 cm above the lowest location in the pre-TAG surface (Fig. 2B and Fig. 16C).

Comparison of pre- and post-TAG images indicates the exposure by TAG of multiple meter-scale rocks at the lowest location in the pre-TAG surface (Fig. 16, D and E). The thruster impingement in this region appears to have scoured away a surficial layer of pebbles and small rocks, exposing and tilting the meter-scale rocks visible in the post-TAG images. Comparison of these rocks to other rocks mobilized by the thrusters suggests the mobility of the rocks appears to depend on the fractional height of the rock above the pre-TAG surface (Fig. 13). Rocks resting on the surface, or nearly so, were mobilized, whereas “iceberg” rocks — those with most of their volume buried — were not ejected.

The presence of ∼1 m rocks at a consistent vertical location in the DTM, seen in two locations, suggests a “basement” layer of ∼1 m or larger rocks, which may have contributed to the change in material properties at depth. The top of this layer is ∼30 cm below the surface at the TAG point, whereas ∼3 m to the west (lowest part of the pre-TAG surface) it is essentially at the surface (see the star in Fig. 16A). This vertical location proved uniquely resistant to thruster-driven excavation and corresponds with a change in the spacecraft acceleration for almost 2 s before the backaway thrusters started. Central mounds in some craters on Bennu suggest the presence of a stronger subsurface (Daly et al. 2020b), and Ryugu studies pointed to a stronger subsurface below a weaker upper layer (Arakawa et al. 2020; Kadono et al. 2020; Ogawa et al. 2022).

#### Intermediate Finer-Grained Layer

The instrument optics, which were pointed towards the surface during TAG, experienced dust deposition from the sampling event (Lauretta et al. 2022). In our simulations, we find that the convergence of the four thruster plumes underneath the spacecraft created a recirculation regime (Fig. 9B), which could sweep small particles underneath and within 1–1.5 m from the TAG point up towards the spacecraft. The presence of dust-sized particles in the TAGSAM ejecta that were available to be recirculated when the thrusters started indicates that dust was ejected relatively late in TAGSAM excavation, TAGSAM deeper than 0.3 m, ∼6 s after first contact (movie S1). This is consistent with recent reports of near-subsurface finer-grained layers on Bennu (Bierhaus et al. 2023) and Ryugu (Cho et al. 2021).

#### Lateral Variation in the Surface Layer

Surface erosion occurred across the TAG site (Fig. 16A), corresponding to a range of thruster PPS (Fig. 16B). Across much of Hokioi crater, the upper ∼20 cm of regolith was mobilized by a broad range of thruster pressures. Previous analysis of the TAG point (Walsh et al. 2022) found a density in the upper 10–20 cm that is just 37–50% of Bennu’s ∼1200 k/m^3^ bulk density (Scheeres et al. 2019), with corresponding high porosity. This condition provides less impedance to expanding thruster gas impinging from above and is consistent with susceptibility to thruster-driven erosion. However, as we described in the context of Fig. 16 the lowest parts of the pre-TAG Hokioi crater floor demonstrated resistance to thruster-driven erosion, meaning the mobility of this upper layer is not universal.

### Relative Effects of TAGSAM and the Thruster Plumes

The correlation of TAGSAM and thruster effects, separated in time, enable us to distinguish their relative effects. From observations of TAGSAM at contact and spacecraft accelerometer data (Walsh et al. 2022; Lauretta et al. 2022), it is apparent that TAGSAM initially released its nitrogen gas in the near subsurface (Fig. 2), and downward spacecraft motion continued throughout gas release. In conjunction with near-zero lateral motion, the release of TAGSAM gas was constrained to a column in the subsurface defined by the TAGSAM diameter and depth of penetration. This geometry is consistent with diffused gas disruption: gas pressure is high around TAGSAM, and decreases radially away, including up towards the surface. Material mobilization occurs in the direction of pressure gradients, pushing material radially away from TAGSAM, which causes ejection of material at the surface. This behavior was observed in vacuum lab testing of TAGSAM with low-density materials (Bierhaus et al. 2021).

The majority of the TAGSAM gas was released over ∼5 s (Fig. 2A), and the deposition of the gas occurred over TAGSAM depths from several centimeters to ∼0.45 m (Fig. 2B, Fig. 16C). The gas pressure and mass-flow rate decayed exponentially, with much less energy deposited at greatest depth than at initial gas release. The expansion of the gas through the regolith was rapid (Bierhaus et al. 2021). When the sample gas was expended, the TAGSAM crater growth followed classical crater behavior, with expansion slowing (Fig. 6) as the energy and momentum are deposited into an increasing volume.

**Fig. 17:**
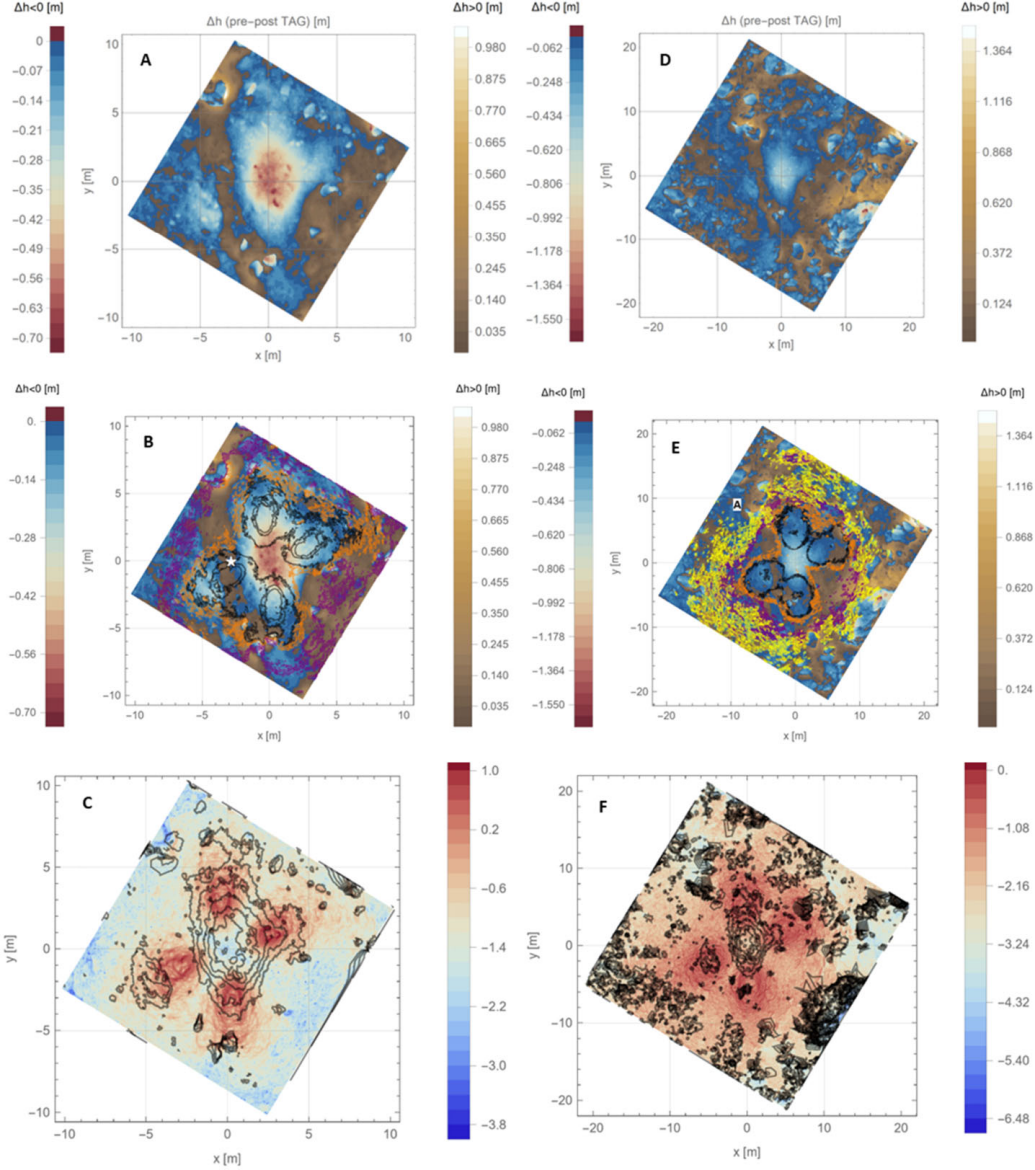
Spatial association between surface erosion and thruster-plume magnitudes. Panels (A) to (C) are from the simulation at the start of the backaway maneuver; panels (D) to (F) are from the simulation at the end of the backaway. **(A)** The $\Delta $h DTM (change in height after TAG) for the backaway-start simulation, rendered in two color gradients: a red-to-blue color gradient for erosion and a brown-to-white gradient for deposition. **(B)** Contours of PPS magnitudes overlain on the DTM in (A). The purple, orange, and three black contours are 0.01, 0.05, 0.1, 0.5, and 1 Pa, respectively. The white star is the lowest location in the pre-TAG surface. **(C)** The underlying plot is the log of the PPS gradient (log(dPPS/ds)). The black contours are for surfaces that experienced net erosion (the blue-to-red regions in (A)) in steps of 10 cm. The deepest contour is the most central. **D)** The same as (A), but for the backaway-end simulation. The ranges of the color gradients change because the expanded surface area includes a different range of $\Delta $h values. **(E)** The color contours have the same values as in (B); the additional yellow contour is 0.005 Pa, and the one fewer black contour is the absence of any values $\geq 1\text{ Pa}$. **(F)** The same as (C) but for the backaway-end simulations. Note the change in scale represented by the color gradient

In comparison, the pressure from thrusters was sustained for 25 s, delivered from above the surface, and distributed over an area of ∼100 m^2^. The thruster gas accumulated rapidly and built a stagnation region on the surface (Fig. 9 and Fig. 10), which provided a constant supply of gas that was injected into the subsurface. This condition pressurizes void space between particles and mobilizes them (Mehta et al. 2013). The pressurized regions were replenished with additional gas from the thrusters for the entirety of the backaway burn. The peak pressure dropped by a factor of ∼7 (Fig. 17) as the spacecraft range increased, compared to ∼two orders of magnitude reduction in the TAGSAM pressure. Unlike TAGSAM, which penetrated into the subsurface with the mass of the spacecraft behind it, the only momentum driving deeper penetration was that of the thruster gas. The peak PPS value and peak gradient are 4.2 Pa and 20.7 Pa/m at backaway start, respectively, and ∼0.6 Pa and 2.7 Pa/m at backaway end.

Although our simulations do not model movement of the surface in response to the thruster plumes, we can estimate where thrusters mobilized material by comparing the modeled thruster pressure and shear with areas that experienced erosion, as indicated by height differences in the pre- and post-TAG surfaces (Fig. 17, A and D). Equation (6) (Metzger 2024a) leverages properties of the gas above the surface to estimate erosion of the surface. Our simulation data, by virtue of using the DTM in conjunction with accurate spacecraft thruster models and relative geometry, enable us to evaluate the plume environment above the surface, as well as the thruster-driven environment at the surface itself. Because the thruster erosional processes depend on the magnitude of the pressure and shear, as well as the gradient acceleration, we consider both the magnitude of the PPS (Fig. 17, B and E) and the gradient (Fig. 17, C and F) for the backaway-start and backaway-end simulations.

PPS values of > 0.05 Pa and dPPS/ds values > 0.5 Pa/m approximately encompass all regions with erosion > 10 cm (the vertical resolution of the $\Delta $h DTM) within ∼6 m of the TAG location (Fig. 17, B and C). Erosion may have occurred at values as low as 0.005 Pa within the extended TAG region (Fig. 17E); however, this result is ambiguous owing to the uncertainty of the DTM, especially the inability to resolve height changes over very short length scales (e.g., boulder edges, Palmer et al. 2024). Most of the deeper, thruster-driven erosion close to the TAG location likely occurred early in the backaway burn when the surface experienced peak and near-peak pressure and shear values. The PPS contours that correspond to less erosion persist through the end of the backaway burn. All $\Delta $h < −10 contours beyond ∼7 m from the TAG location correspond to discrete, smaller rocks or the faces of larger boulders. Pre- and post-TAG imaging confirms that some of these rocks were ejected (Fig. 12 and Fig. 13).

When the thrusters first turned on, much of the area underneath the thruster stagnation region was the pre-existing asteroid surface; however, immediately underneath the spacecraft and around TAGSAM was the expanding TAGSAM-driven crater. As previously discussed, at the time the thrusters turned on, the TAGSAM crater was ∼0.5–0.7 m radius and expanding at no more than a few centimeters per second (Fig. 6). Though interior to the peak pressure and shear values from the four thruster footprints, the TAGSAM crater resides within the 0.05-Pa pressure contour (Fig. 17B), which corresponds to erosion elsewhere in the $\Delta $h DTM. Thus, at thruster initiation, the initial condition of the surface for the thruster plumes included the TAGSAM crater interior and surrounding regions. In the areas where the thruster footprints fell outside the expanding TAGSAM crater, the thruster excavation started from the pre-TAG surface; thus, outside ∼0.7 m radius, the TAGSAM crater expanded into surface that had already experienced erosion. Because the thrusters had already removed material before the TAGSAM crater’s arrival, the crater likely was able to expand further than it otherwise would have because it was excavating less volume than existed in the pre–thruster-fire surface. The thrusters alone removed material in regions outside the maximum extent of the TAGSAM crater.

**Fig. 18:**
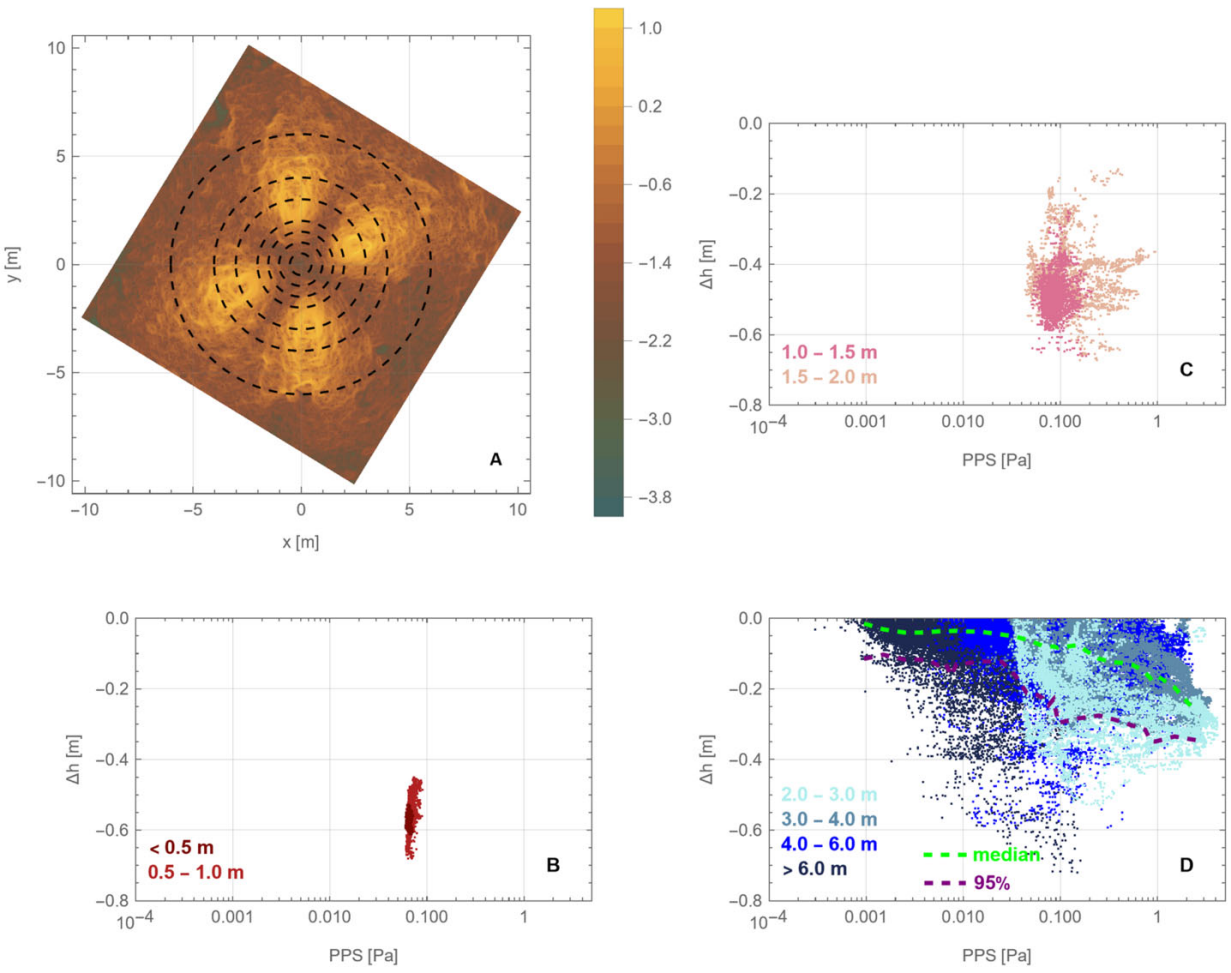
Relationships between distance from the TAG point, thruster PPS, and erosion. **(A)** The thruster PPS gradient (Fig. 18C), replotted with a different color scale and superimposed with circles whose radii correspond to the distances in panels (B) to (D). The plots in (B) to (D) compare regions with $\Delta$h < 0 and thruster PPS, separated by radial distance (indicated by color) from the TAG point. **(B)** Distances < 1 m from the TAG point correspond to TAGSAM-dominated excavation because the $\Delta $h magnitude is independent of thruster PPS. **(C)** Distances of 1–2 m correspond to superimposed TAGSAM and thruster effects. **(D)** Distances beyond 2 m correspond to thruster-dominated erosion. The dashed green line is the median $\Delta $h values for the distances shown, and the purple line denotes the 95% of the $\Delta $h values, i.e., 95% of the $\Delta$h values at a given distance are above this line. The lines demonstrate a systematic increase in erosion with increasing PPS

The relationships between radial distance from the TAG location, the magnitude of erosion ($\Delta $h < 0), and thruster PPS provide evidence for the transition between TAGSAM-dominated erosion and thruster-dominated erosion (Fig. 18). Because 10 cm above the lowest location pre-TAG responds anomalously to the thrusters, we consider data for locations with pre-TAG heights > 10 cm. For radial distances ≤ 1 m from the TAG location, the erosion depth is independent of PPS (Fig. 18B), indicating TAGSAM-dominated excavation. Radial distances between 1–2 m are a transition zone in which thruster PPS increases (Fig. 18C). Beyond 2 m there is a correlation between erosion and PPS magnitudes (Fig. 18D), indicating thruster-dominated erosion beyond 2 m radius, consistent with our other constraints above.

### Final Dimensions of the TAGSAM Crater If the Thrusters Had Not Fired

Our observations provide two new insights for the TAGSAM-driven crater. The first is for the expected shape of a TAGSAM-only crater, the second is for the TAGSAM-created crater in the absence of augmentation from the thrusters.

The distance of the ejecta edge from the TAG location varies depending on the azimuth of the radial profile (Fig. 5A). Notably, the ejecta edge visible in the NavCam images suggests an elliptical shape. Variability in the TAGSAM crater radius derived from images (Sect. 2.3.2) is correlated with height variability in the pre-existing surface and thus can be explained by conservation of momentum as the expanding pressure wave moves into an asymmetric distribution of mass. The pressure wave moves farther in directions where there is less mass (i.e., northward and southward), and moves less far in directions where there is more mass (east), or resistance to erosion (west). In other words, the pre-existing ellipticity of the “hill” at the contact point (Fig. 1) corresponds to an elliptical feature in the $\Delta $h DTM (Fig. 17). Thus, had the TAGSAM crater continued without thruster-plume effects, the final crater likely would have been elliptical, elongated north-to-south and narrower east-to-west.

The average pre-thruster-activation expansion rate of the inner edge of the TAGSAM-driven ejecta is consistent with a 3-m-diameter final TAGSAM crater (Fig. 7), if that crater were not modified by the thrusters. Data points above and below the line correspond to the ellipticity of the inner edge. Further, the diameter of a gravity-scaled impact crater, approximating TAGSAM as an impactor with kinetic energy equivalent to the potential energy of the pre-released gas, is ∼3.2–3.3 m (Fig. 8). These completely independent estimates are within ∼10% of one another. Of course the impact-crater analogy assumes a circular feature, yet the consistency of the two results using independent techniques provide confidence in the size of the TAGSAM-only crater.

### Regions with Net Accumulation

Our analysis identifies why the $\Delta $h DTM (Fig. 17, A and D) shows several areas of net accumulation after TAG. The regions with the most accumulation are surfaces with pre-TAG heights that increased radially away from the TAG point, including the region to the east and southeast of Hokioi crater, in the direction of Mt. Doom and adjacent high-standing rocks on Hokioi’s rim.

**Fig. 19:**
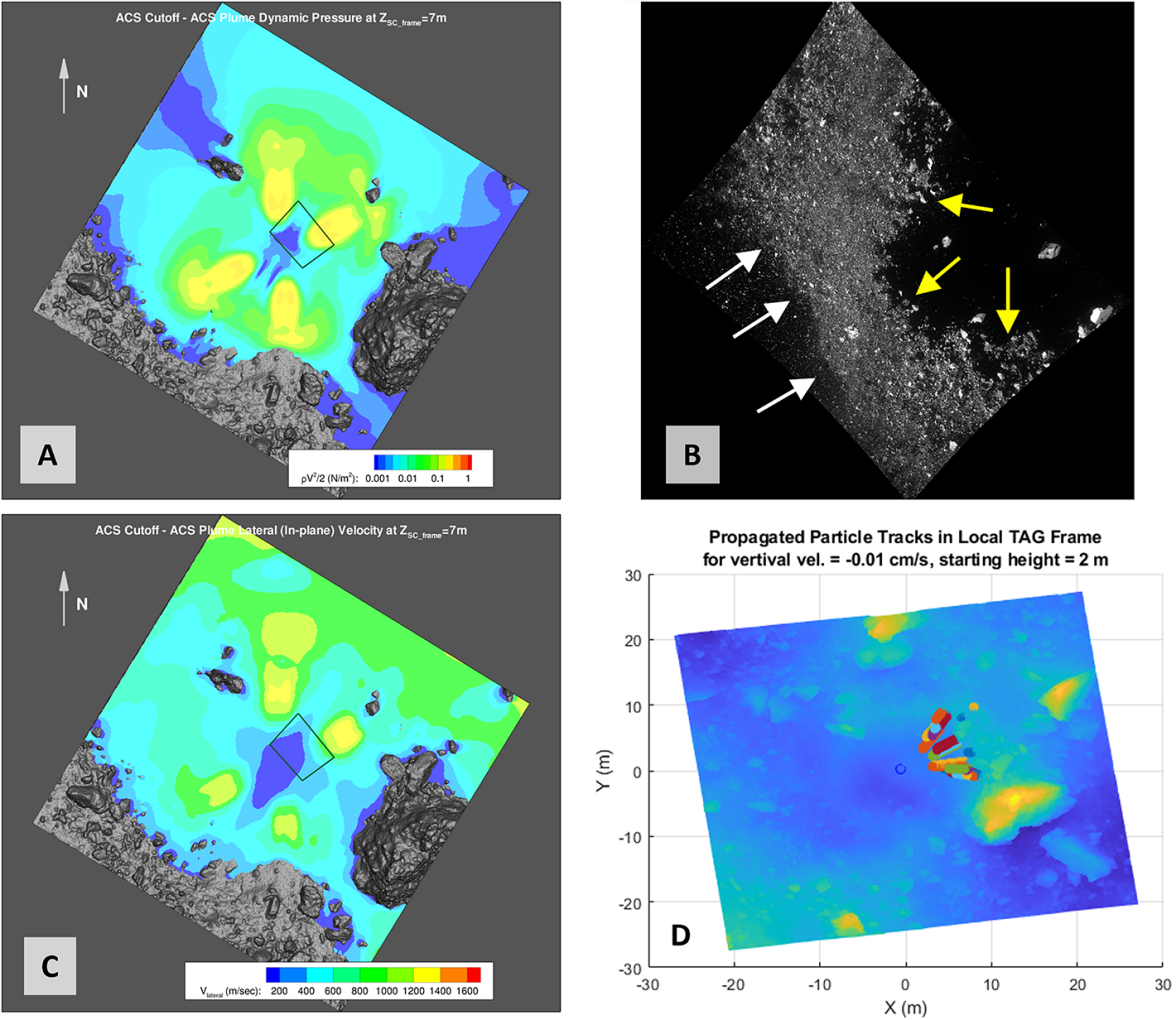
Dynamic pressure and particle motion. North is up, and east is to the right. **(A)** Dynamic pressure, in log scale, of the thruster plume above Bennu’s surface at the end of the backaway maneuver. The 7 m in the plot label refers to the height of this data “slice” in the spacecraft frame, which is 1.3 m average height above the surface. The black trapezoid is the NavCam FOV at the end of the backaway maneuver. **(B)** NavCam image 20201020T215021S680, taken at the end of the backaway maneuver. The yellow arrows indicate the boundary of material redirected by the thruster plumes (see yellow region inside the NavCam FOV in (A)). The white arrows indicate the inner edge of the TAGSAM ejecta, not caused by the thrusters. **(C)** Lateral velocity component of dynamic pressure from (A). **(D)** The colored tracks are the estimated trajectories (Supplementary Materials) for particles distributed across the edge of ejected material indicated by the yellow arrows in (B). The small, isolated blue circle is the TAG site. The background is the 3D topographic height (the same data as Fig. 1 A and B, with a different color scale)

Ejected material visible in the NavCam images throughout the backaway was subject to significantly varying dynamic pressure (Fig. 19A, movie S1) from the thrusters. By comparing the dynamic pressure of the thruster plumes with projected NavCam images, we find good agreement between higher-dynamic-pressure regions and trajectory modification of the in-flight ejected regolith (Fig. 19, A and B, Sect. 2.6, movie S1), and conclude that dynamic pressures greater than ∼0.1 Pa are sufficient to redirect ejecta (Fig. 19, A and B). A very steep gradient in dynamic pressure magnitude corresponds to the boundary between redirected and non-redirected material, which supports the interpretation that the thruster plume created a nearly discrete boundary between redirected and non-redirected material.

The lateral component of dynamic pressure varies across the TAG site, influenced by TAG site topography (Fig. 19C). Particles distributed along the shear boundary (Sect. 2.6 and Supplementary Information) of the thruster plumes (which appear in the steep gradient from high to low dynamic pressure) land just to the east of the TAG site, where the eastern rim of Hokioi crater and the boulders on it introduce a steep increase in height (Fig. 19D), consistent the appearance of “new” particles in the post-TAG images at those locations (Fig. 12D). Given the bulk of the ejecta behind these tracer particles follows a similar trajectory, material deposition occurs at the base of the rim and tall rocks, explaining the net accumulation of material in the $\Delta $h DTM. In comparison, there are few regions in the western TAG area with accumulation > 10 cm. The Hokioi rim heights are lower to the west, and there are fewer topographic barriers to ejected material. In addition, less mass is available to mobilize to the west because of the decreasing pre-TAG surface heights (Fig. 1).

We conclude that the distribution of accumulated material was the result of two factors: (i) the availability of mass available to be mobilized in a given direction, and (ii) the presence or absence of topographic barriers to ejected material. The generally eastward directions had more mass available for ejection and greater topographic barriers to that ejecta compared with westward directions (Fig. 1, Fig. 19 A and C).

### Constraints on Cohesive Forces

The thruster-plume simulations provide a novel method to constrain the cohesive forces on Bennu. Comparing the material mobilization by the thrusters against particle weight alone provides an opportunity to evaluate cohesive forces. This approach considers the ensemble of the DTM rather than focusing on individual rocks, for two reasons: (i) the spatial resolution of the mesh is insufficient to capture accurate shape information on discrete particles to derive correspondingly accurate thruster effects (with some exceptions, one of which we discuss below), and (ii) the DTM has lower accuracy at locations with rapid changes in height (e.g., rock faces). Although the thruster modeling correlates well with mobilized particles — that is, the rocks that have modeled high pressure from the thrusters are ejected (Fig. 10, Fig. 13) — the accuracy of the specific, integrated pressure on each rock is limited by the mesh accuracy. However, the DTM is sufficiently accurate to capture thruster-surface forces at length scales longer than individual rocks.

**Fig. 20:**
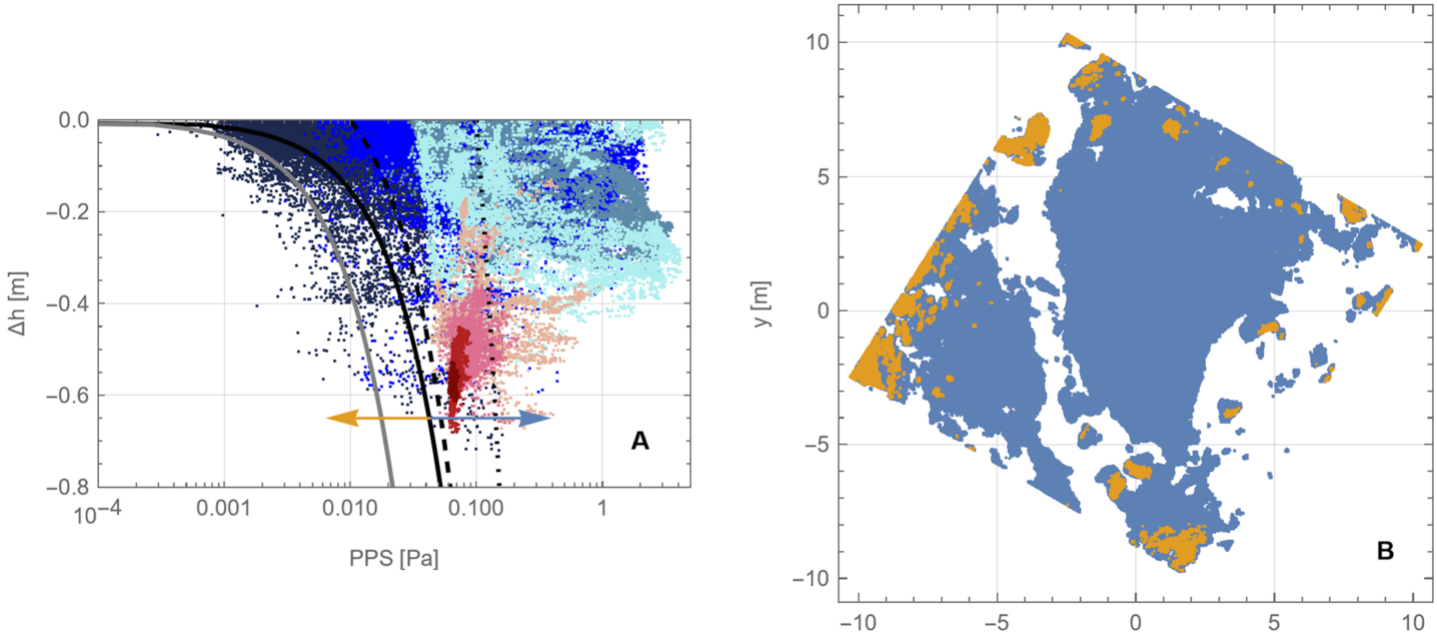
Constraints on cohesive forces and bulk density at TAG site. **(A)** Panels B to D from Fig. 18 combined, with superimposed curves. The two solid curves correspond to lithostatic pressure on Bennu as a function of depth for two bulk densities: $\rho \sim 1200\text{ kg}/\mathrm{m}^{3}$ (black curve), the global Bennu bulk density (Scheeres et al. 2019), and $\rho = 500\text{ kg}/\mathrm{m}^{3}$ (gray curve), the estimated bulk density of the upper $\sim 10\text{ cm}$ (Walsh et al. 2022) at the TAG point. The dashed and dotted black curves are the $\rho =1200\text{ kg}/\mathrm{m}^{3}$ case with the addition of 0.01 and 0.1 Pa cohesion, respectively. The blue and orange arrows correspond to two populations of data. The blue arrow points to a regime where the thruster PPS value was sufficient to overcome lithostatic pressure if $\rho =1200\text{ kg}/\mathrm{m}^{3}$; the orange arrow points to a regime where the thruster PPS does not exceed the lithostatic pressure for $\rho =1200\text{ kg}/\mathrm{m}^{3}$. **(B)** The spatial locations of the two data types defined by the blue and orange arrows in (A). All orange data correspond to the tops or edges of rocks, or steeper vertical locations. These are either low PPS locations on a rock whose “windward” face experienced high PPS, or regions with rapid height change, which is not as accurate in the DTM (Palmer et al. 2024)

We compare the thruster PPS against lithostatic pressure for two different bulk densities (Fig. 20A). In the case of zero cohesion, the only resistance to mobilization from the thrusters is gravity. We find that thruster PPS is sufficient to cause nearly all erosion that occurred across the TAG site (Fig. 20A). There are some facets for which the thruster PPS falls below the lithostatic pressure for Bennu’s bulk density and/or a lower near-surface density. However, there are two effects to consider in these cases (Fig. 20B): (i) all cases are at the edges of rocks or at rapid changes in heights, and thus have lower accuracy in the DTM, and (ii) these often are the leeward side, or top, of particles that have higher pressure on the windward side. The pressure-gradient force (described earlier) would mobilize these particles due to the contrasting higher- and lower-pressure across the windward and leeward faces of these particles.

If there were non-gravitational forces that created retention effects greater than that of lithostatic pressure alone, the pressure needed to mobilize the regolith would be shifted to higher values (see dashed and dotted curves in Fig. 20A). The presence of surface elements that were mobilized at lower pressures indicates that such cohesion did not exist. Because the spatial resolution of the DTM limits our ability to constrain arbitrarily small pressures (because of the minimum surface facet area), and because we do not model the full two-phase flow between the thruster gas and regolith, we cannot conclude there was zero cohesion; however, any cohesion that exists is very small (less than ∼0.1 Pa). This finding corroborates previous studies of the TAG region (Barnouin et al. 2022; Walsh et al. 2022).

The thrusters ejected a ∼1 m rock (see the rock with large pressure values in the southern thruster footprint in Fig. 10A) about 12 m (Lauretta et al. 2022), and though the rock shape suffers the same accuracy constraints as other short-wavelength features, it is sufficiently resolved by the DTM (1172 facets) that we derive a net force from the thrusters of 0.51 N. For a volume of 0.38 m^3^ (derived from the resolved feature in the shape model), density of 1800 kg/m^3^ (approximately the highest stone density from the Bennu sample reported in Lauretta et al. 2024) and Bennu surface gravity of $5.42{\times} 10^{-5}\text{ m}/\mathrm{s}^{2}$ (Daly et al. 2020a), the rock weight is ∼0.04 N. We do not know the full shape of this rock, and it could have a higher density. If we conservatively assume that the volume and density are each twice as large, the weight would be 0.16 N; in this bounding case, the thruster force is still ∼3.2 times larger than the weight. The mobilization of this rock demonstrates how the dynamics in a micro-gravity environment bely terrestrial, 1-g intuition: relatively small thruster pressures can mobilize a single rock with a mass of hundreds of kilograms. The thruster force will be increasingly effective at mobilization as particle size decreases because of increasing area-to-volume ratio, in the case of low or no cohesion, which seems to be the case for regolith in the TAG region (Walsh et al. 2022; Lauretta et al. 2022; as well as our conclusions above).

### Erosional Processes and Characteristics of the Final TAG Erosion Region

The thruster simulations and analysis of TAGSAM-driven ejecta provide a new perspective on the TAG erosion region, and the processes that excavated regolith.

**Fig. 21:**
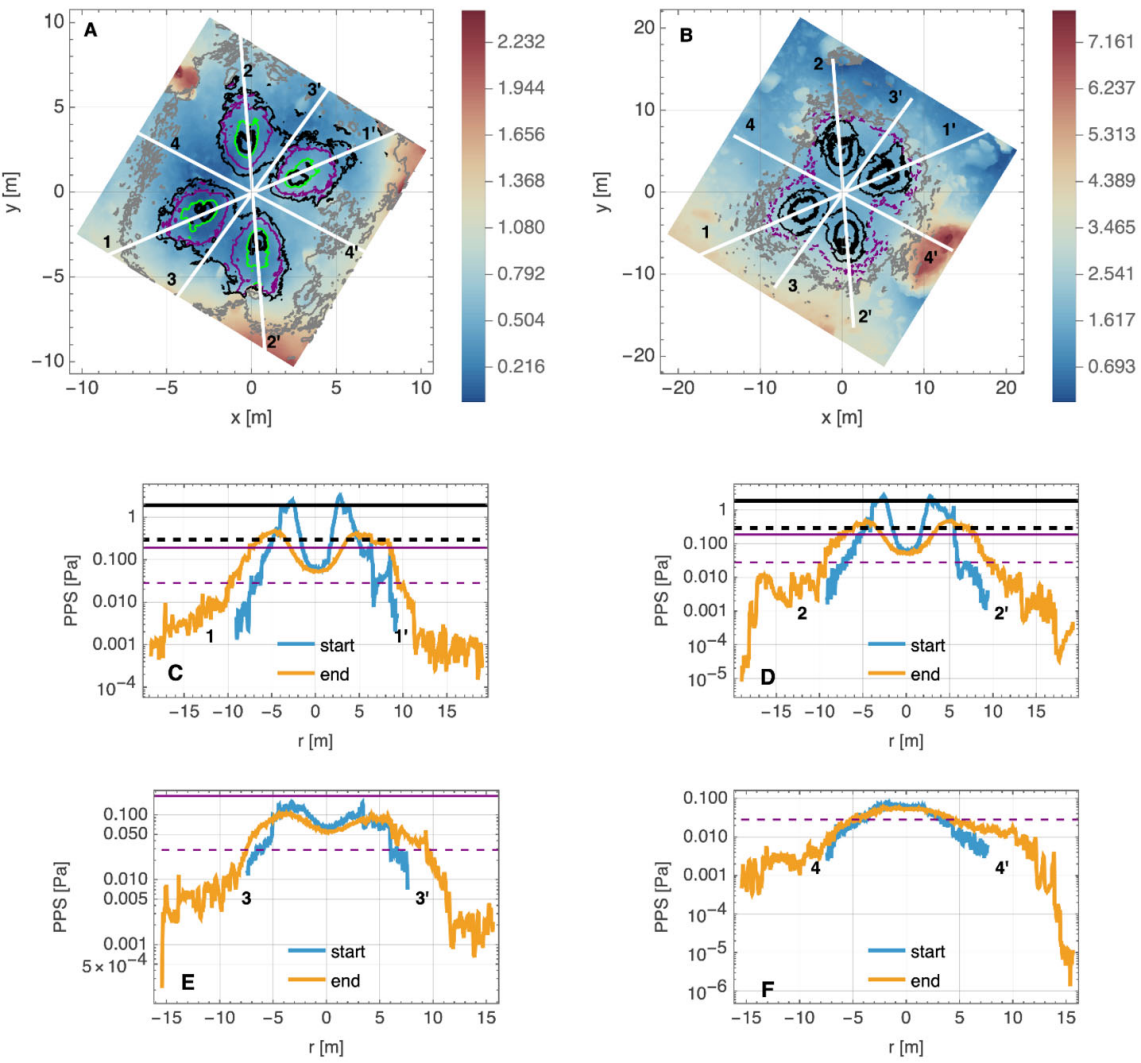
Thruster profiles from both simulations. **(A)** and **(B)** are the backaway start and end simulation domains, respectively. The background color for each is the geometric height of the domain. Note the change in scale between the two panels. To enable comparison of both absolute and relative values of PPS we select fixed and relative values within each domain based on profiles that sample the same plume geometry between each simulation. Each has four white lines that are profiles across four different PPS profiles from the simulation. Profiles 1 and 2 bisect the four main footprints of the thrusters. Profile 3 bisects the shorter dimension between the plumes, and profile 4 bisects the longer dimension between the plumes. In each plot the outer gray contour is for 0.01 Pa, and the thin black line is for 0.1 Pa. The green contour in (A) is for 1 Pa, which is not present in (B) because the maximum value in B is 0.6 Pa. The purple contour is 5% of the maximum value in each simulation, or 0.20 Pa in (A) and 0.03 Pa in (B). The thick black line is 50% of the maximum value in each simulation, or ∼2.0 Pa in (A) and 0.3 Pa in (B). Panels **(C)** through **(F)** plot the PPS profiles from each simulation. The thick black and purple lines correspond to the 50% and 5% contours in (A), and the dashed versions of these lines correspond to the 50% and 5% values in (B)

The backaway maneuver lasted ∼25.5 s (Fig. 6). At the start of the maneuver the thrusters were ∼3.14 m from the surface, then descended another ∼5 cm before halting then reversing the downward motion of the spacecraft. The thrusters were ∼6.6 m from the surface when the backaway maneuver ended. As the spacecraft distance increased from the surface the peak PPS decreased while the footprints expanded; thus, the PPS on a given surface element changed as a function of time and location. We consider the PPS evolution across four profiles, two of which sample the axes that bisect the thruster plume cores, and two of which sample the minimums between the plumes (Fig. 21). Dimensions of roughly symmetrical profiles can be characterized in terms of where the values are, e.g., 50% of the maximum. In our simulations we find that 50% of the maximum value spans a relatively small distance, ∼0.6–0.7 m, within the plume core for the backaway start because the peak values are sensitive to small-scale topographic variations (Fig. 21A). The 5% of maximum PPS contour for the backaway start is between 3.6 and 4.7 m, with the longest case associated with the plume pointing approximately south (Fig. 21A). For the backaway end, the maximum extent of the 50% contour is between 3 and 4.5 m (Fig. 21B). The maximum extent of the 5% contour for the backaway shifts from four separate contours around each footprint to a single contour that wraps around the impingement region, significantly increasing the lateral extent to ∼19 m in the longest dimension.

**Fig. 22:**
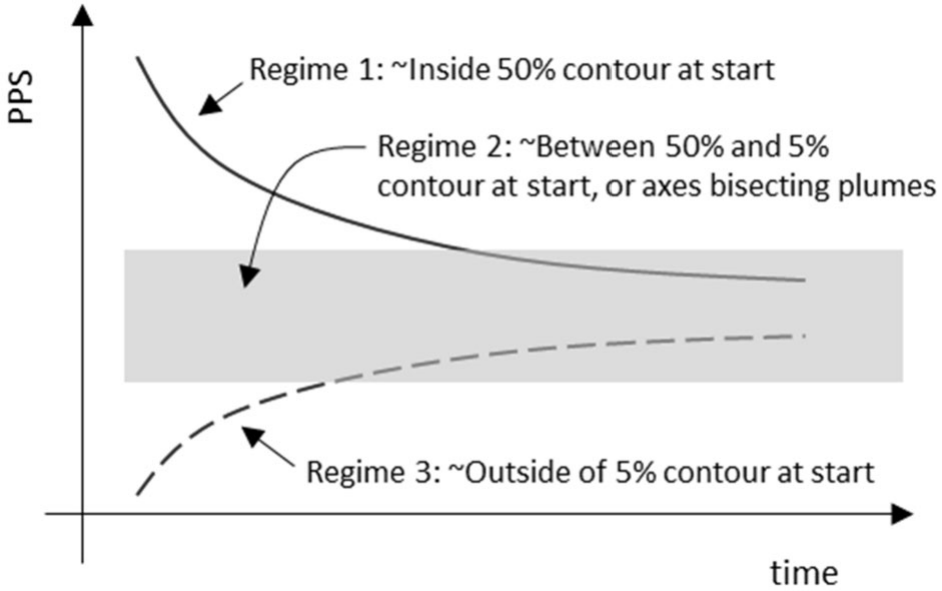
Schematic of PPS time-dependent profiles during the backaway maneuver. We generalize the time dependency of PPS profiles into three regimes: one in which the PPS systematically decreases (solid black line), one in which the PPS may increase or decrease over time by approximately tens of percent (light gray box), and one in which the PPS systematically increases

Based on the behavior of the plume profiles in Fig. 21, we create a simplified schematic of the PPS profile as a function of time, depending on initial location relative to the backaway start (Fig. 22). Using only relative magnitudes and approximate locations, there are roughly three types of PPS time-dependent behaviors. The first corresponds to PPS that systematically decreases in time, and occurs in four separated regions inside the higher-PPS contours of the backaway start. The second regime is a relatively narrow regime when the PPS is approximately constant or changes of-order tens of percent during the backaway maneuver; this occurs in the intermediate- and lower-PPS contours of the backaway start individual footprints, or in the regions between the footprints. The third regime consists of regions that experience a systematic increase in PPS, which occurs at the fringes of the ∼5% PPS backaway start contour and in between the footprints. Regime 3 occurs because the backaway end thruster footprints shift radially outward relative to the backaway start, exposing more distant regions from the TAG location, which had low PPS at backaway start, to higher PPS.

We have insufficient data to characterize whether the maximum erosion depth for a given PPS value occurred. Low-gravity experiments reported in, e.g., Metzger (2024a) and LaMarche (2013) indicate that the time to reach maximum erosion depth increases for decreasing surface gravity, and maximum depth for lunar-g experiments occurs between several to tens of seconds, depending on the material. These experiments also indicate that the thruster excavation width continues to grow over longer time scales than the depth. Given that Bennu’s surface gravity is smaller than that of the Moon by a factor of > 30,000, and the OSIRIS-REx backaway maneuver lasted ∼25 s, it is reasonable to assume that the thrusters did not reach maximum erosion depth or width, at least in those locations where an easily mobilized layer remained.

**Fig. 23:**
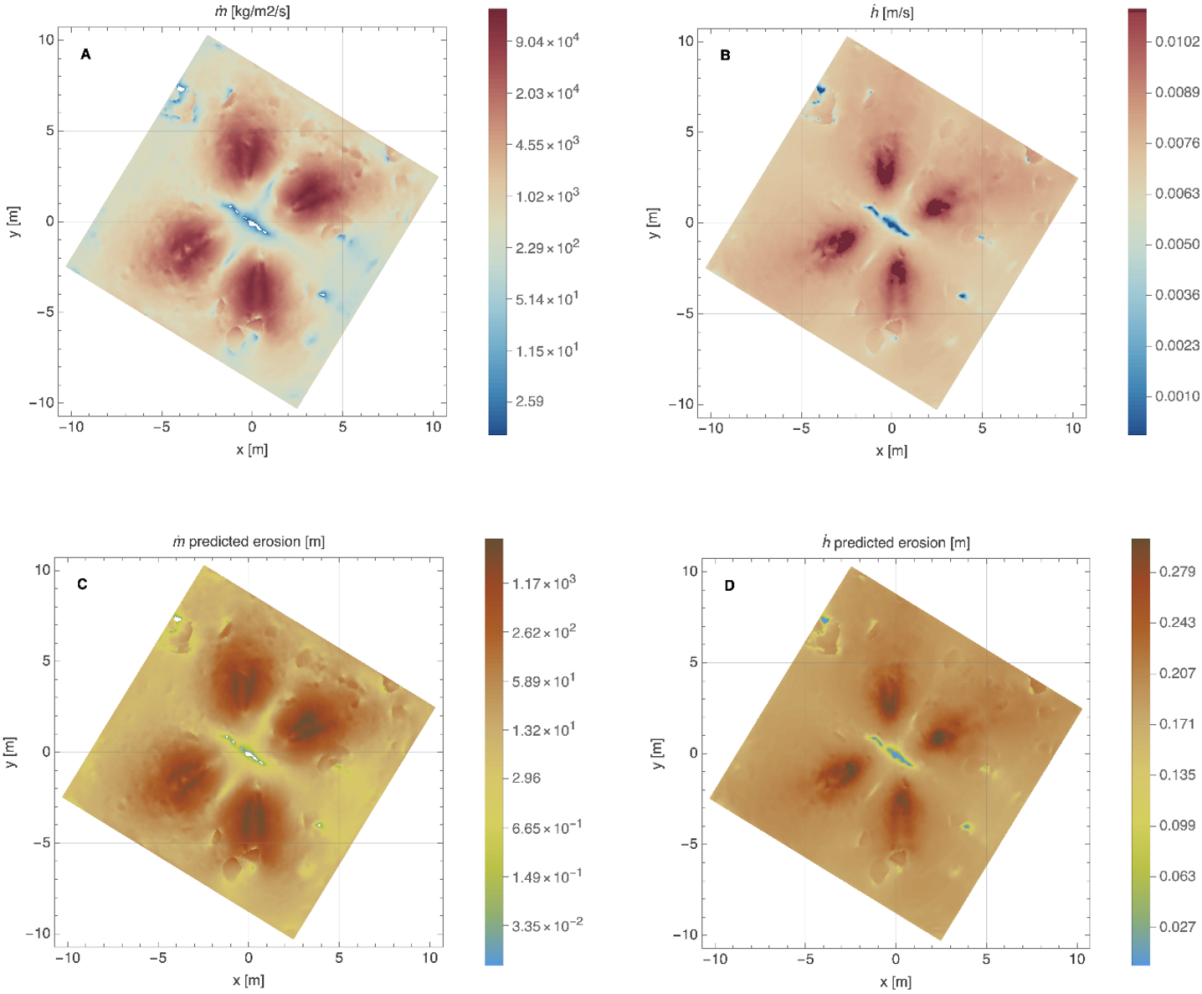
Comparison of the erosion rate from Eq. (6) and our modified form in Eq. (7). **(A)** Calculated values for $\dot{m}$ (units of kg/m^2^/s) for Eq. (6) from the backaway start simulation. **(B)** Calculated values for our modified expression of erosion rate, $\dot{h}$ (units of m/s), see Eq. (7). **(C)** The corresponding total erosion, in meters, during the back-away maneuver calculated from (A). Local erosion depths exceed hundreds of meters within the core plume, and there is significant variation in erosional depths; this result is orders of magnitude too large to be consistent with the observations. **(D)** The corresponding total erosion, in meters, during the back-away maneuver calculated from (B). Though the derived values differ from observed values by tens of percent to a factor of a few, the results are much closer to observations, including a smaller variation between inside and outside the thruster footprints

We return to Eq. (6) to estimate the distribution of mass erosion rates. As noted in Sect. 2.4.1 the available in-flight data prevent us from following an equivalent quantification described in Metzger (2024a) and (2024b), though our simulations provide a methodology to derive functionally similar values (see Supplemental Information). Using Eq. (6) we find mass erosion rates that predict total erosion depths that far exceed those observed (Fig. 23). Though a detailed derivation is outside the scope of this paper, we propose one explanation: it appears that Equation (6) was derived assuming that the mass loading of already mobilized regolith does not affect the efficiency of the thruster plume to mobilize more regolith. In the case of the altitudes considered for the derivation (Apollo lunar module altitudes > 15 m) this indeed may be the case. Morris et al. (2015) simulated Apollo-like module at lower altitudes, and in a 5 m altitude case found that dust loading does affect the thruster erosion efficiency (their Fig. 12). Essentially the dust loading decreases the dynamic pressure of the gas, which decreases erosion. To incorporate the mass loading effect into Eq. (6) we use a simplified methodology (see Supplemental Information) to derive:

7 $$ \dot{h} = \frac{\varepsilon \left ( \frac{1}{12} \rho _{0} v_{0}^{2} \overline{v_{T}} - E_{th} \right )}{\rho _{b} g \left \langle D \right \rangle +\alpha +c \rho _{0} v_{0}^{2}} $$

where all the terms are the same as Eq. (6), except that we replaced $\dot{m}$ with $\dot{h}$, an erosion rate (change in height) in m/s, and we included $c \rho _{0} v_{0}^{2}$ in the denominator to account for energy transferred from the plume to regolith kinetic energy. The parameter $c$ is a constant that scales the deposition of plume energy into the regolith kinetic energy. We use an area-weighted average erosion depth of 20 cm, across the backaway start domain, with the ∼25 s duration of the backaway maneuver to estimate an average $\dot{h} =0.008\text{ m}/\mathrm{s}$; substituting values (see Supplemental Information) for the other parameters, we find c ∼ 26.

The resulting spatial distribution of predicted erosion depths via our modified formulation is within tens of percent of the observed values, and the dynamic range of the depths more closely matches observations; in this sense the erosion rate from gas dynamics near the surface predicts erosion patterns similar to the PPS values at the surface. On the other hand, this methodology does not provide the actual pressure and shear on discrete rocks for mobilization in the way that the PPS (related to total energy available to do work) and PPS gradient (which is related to the acceleration magnitude) do (Fig. 17). The magnitude of height variation of a rubble-pile surface may challenge the use of a single value such as 〈D〉 in Eqs. (6) and (7). More research remains to develop generalized modeling techniques that capture erosion dynamics for regoliths that span higher-gravity and high cohesion environments like the Moon, and lower-gravity and low-cohesion environments on small bodies (e.g. Bennu).

The total TAGSAM-plus-thruster erosion region (Fig. 17 A and D) is characterized by (i) a deep central area started by TAGSAM, then expanded by both TAGSAM and the thrusters, surrounded by (ii) a broader and shallower region that was excavated by thrusters alone — as much as 30 cm erosion within 6 m from the TAG location, and 10–20 cm for regions further than 6 m. The transition from continuous to irregular erosion, with increasing distance from the TAG point, prevents identifying clear boundaries to the “TAG crater”, motivating our use of the phrase “TAG erosion region”.

The distribution of thruster pressure, and relative values of mass erosion rates, generally correlate with regions of surface erosion, including locally high pressure values where most of the thruster plumes impinge on the surface, and on the ∼1 m rock that was ejected from Hokioi by the thrusters. Due to the flow-field behavior (e.g. Fig. 9b), the spatial distribution of surface pressure (e.g. Fig. 10), and the nature of gas expansion against a porous surface in a vacuum (e.g. Fig. 11), we conclude that there were two dominant processes induced by the thrusters to mobilize material: viscous erosion and diffusion-driven flow. The ejection of surface rocks several meters from the TAG point indicates that viscous erosion played a role, shearing surface particles into space. An additional mechanism is responsible for material excavation below the surface shear layer. Diffusion-driven flow occurs when stagnation pressure pumps gas into the subsurface (Metzger et al. 2021). The presence of a broad, stable stagnation region for the 25-s backaway duration, given that Bennu is a porous body (Barnouin et al. 2019), especially in the upper ∼10 cm (Walsh et al. 2022), suggests that diffusion-driven flow was an excavation mechanism for the thrusters.

## Discussion and Conclusions

The response of Bennu’s surface and near subsurface to the OSIRIS-REx thrusters enable us to reconsider earlier interpretations of the TAG event, with a possible explanation for such a low cohesion. In addition, the significant mobilization of the surface in response to the thrusters reveals a novel approach to probing surface and subsurface properties without direct contact.

### Updates to Previous Analysis of the TAG Event

To estimate the size of the TAGSAM-only crater, Lauretta et al. (2022) assessed thruster effects on the surface by visual inspection of the $\Delta $h DTM and identifying features that could represent the extent of thruster-plume excavation. Here, we combine state-of-the-art simulations of plume-surface interactions with measurements of the growth of the crater generated by the TAGSAM gas only, enabling revisions to the volume of material excavated by TAGSAM and corresponding interpretations of subsurface structure.

First, we estimate a revised TAGSAM-only excavation volume by assigning all erosion inside 0.7 m radius from the TAG point to TAGSAM, 0.2 m erosion (or less if the erosion for a facet of the DTM is < 0.2 m) to the thrusters from 0.7 m to a given outer radius, and all erosion to the thrusters beyond the outer radius. For an outer TAGSAM crater radius of 1.5 m (predicted by comparing observations of the crater growth with crater expansion models; Fig. 7 and Fig. 8), the revised TAGSAM-ejected volume is 2.5 m^3^. For an outer crater radius of 2.0 m (further expansion of the TAGSAM crater enabled by the thrusters’ prior removal of material), the revised volume ejected by TAGSAM alone is 3.5 m^3^. These values are a factor of several less than the 8.6 m^3^ reported in Lauretta et al. (2022). As a result, the subsurface does not require near-surface densities below that of bulk Bennu to relate the potential energy from TAGSAM gas with the kinetic energy needed to displace the volume. The sub-surface could have a bulk volume consistent with that of global Bennu (1190 kg/m^3^, Scheeres et al. 2019). Our results are consistent with a weak, under-dense ∼10 cm surface layer at the TAG point (Walsh et al. 2022) because that depth is mobilized by low PPS values (Fig. 20A). However, our results also indicate this behavior is not universal and can vary laterally at scales of just a few meters (Fig. 16).

Second, we revise the interpretation of some features in the $\Delta $h DTM. (i) We interpret the approximately elliptical, deep central portion of the $\Delta $h DTM as removal of an elliptical raised mound, vs. the slope-driven collapse of a circular crater into an elliptical one inferred in Lauretta et al. (2022). (ii) We consider the “ridge” (Lauretta et al. 2022) in the western portion of the $\Delta $h DTM as a portion of the surface that resisted erosion, rather than the fall-back of material by surface relaxation. (iii) The multiple, broad regions of excavation outside the central, deep ellipse correspond with thruster PPS contours that could mobilize material, in which case no post-TAG surface collapse is needed to explain areas that lost surface height at greater distances from the TAG point.

Third, we estimate the amount of mass mobilized by the thrusters. The total volume of all facets with erosion in the backaway start simulation is ∼17.1 m^3^. Using the range of estimated volume of TAGSAM ejecta above, 2.5 m^3^ to 3.5 m^3^, the thrusters mobilized 13.6–14.6 m^3^ within this region. The erosion could extend beyond this region, and some areas of accumulation in the $\Delta $h DTM that are near large topographic highs (i.e., close to Mt. Doom) may originally have had some erosion that then turned to net accumulation as ejected material hit Mt. Doom and fell to its base. It is challenging to quantify these cases with only “before” and “after” DTMs; thus, we simply identify the thruster-mobilized volume range as a likely minimum. Metzger (2024b) estimates that the Apollo 16 lander may have excavated between 11 to 26 t, meaning the OSIRIS-REx spacecraft excavated a regolith mass a few tens of percent less than, or comparable to, that of the Apollo landers.

### Cohesion Differences Between Solar System Objects

Bennu’s cohesion in vacuum is lower than that of the lunar regolith (Carrier et al. 1991). Like the Moon, Bennu’s surface is collisionally evolved (Ballouz et al. 2020; Bierhaus et al. 2022), so the divergent cohesive properties may be related to compositional differences, ejecta sorting (the Moon’s much higher surface gravity retains a greater fraction of melted, jagged, fast-moving ejecta), or both. Analysis of the regolith sample returned by OSIRIS-REx (Lauretta et al. 2024) is illuminating the similarities and differences in particle morphologies between the typical jagged nature of a lunar fines (a contributor to lunar cohesion) and Bennu particles.

### Thruster Interrogation of Surface Properties

Our analysis demonstrates that it is possible to use spacecraft thrusters to evaluate regolith properties of small planetary bodies without contacting the surface. The outcome of this interaction depends on the thruster pressure and shear magnitudes across the surface, which are a function of both the spacecraft (thruster properties and thruster geometry, range to surface) and the surface (long- and short-wavelength topography, cohesion, layering). The thruster plumes interacted with orders-of-magnitude more surface area than TAGSAM contacted. The broad range of resulting pressures on the surface provides an inherent range of forces, which can identify threshold(s) of mobilization. Lorenz (2023) found a ∼1 Pa threshold for dust mobilization on Mars, which is at least an order of magnitude larger than the minimum value we found for Bennu.

The flow of gas from higher to lower pressure means that thruster effluent not only traveled across the surface and mobilized particles like wind shear on Earth or other planets with atmospheres; it also penetrated a porous subsurface, mobilizing and excavating material. The significant mass loading that can occur may necessitate a more generalized treatment of thruster-driven erosion that includes the condition when the mass ejected by the thrusters will decrease the erosion efficiency of thruster gas impinging later in time. Despite the likelihood that this condition occurred at Bennu, the thrusters mobilized over 13 m^3^ of regolith.

This result is relevant for future spacecraft missions to small bodies, such as OSIRIS-APEX, which is repurposing the OSIRIS-REx spacecraft to explore the near-Earth asteroid Apophis in 2029 (DellaGiustina et al. 2023). The spacecraft will descend close to, but not touch, the surface and use backaway thrusters to mobilize a broad region of regolith. Assuming Apophis regolith has low cohesion (< 0.1 Pa), our results suggest the spacecraft thrusters could mobilize regolith from a range of ∼3–6 m above the surface, affecting a broad surface area at least tens of square meters. The absence of TAGSAM at Apophis simplifies the experiment because the thrusters will be the only disturbing force. We recommend a TAG-like imaging cadence (with the SamCam and NavCam acquiring images at their maximum rate), recording of high-rate inertial measurement unit (IMU) data, and, if possible, a slow rotation of the spacecraft about the z-axis after reaching escape speed. This would permit the NavCam to observe the entire range of surface affected by the thrusters. Because the activity requires minimal fuel in a microgravity environment, the experiment could be repeated at multiple locations across the surface to evaluate variability as a function of observed surface properties. These experiments would be capable of identifying variation in surface and near subsurface properties.

## Supplementary Information

Below are the links to the electronic supplementary material.

Movies generated from SamCam and NavCam image data. The graphic in the upper left is the timeline of the TAG event. The moving red vertical line in the timeline corresponds to the time of each image in the movie. The labeled events in the timeline include: “TAG” for first contact, “Gas” for TAGSAM sample gas release, and “Backaway” for the start of the backaway thrusters. The thick black arrows indicate the directions for Bennu North (N) and the Sun. The left-hand movie consists of SamCam images, showing TAGSAM, the TAGSAM arm, and Bennu’s surface. The center movie consists of static images extracted from the Bennu basemap (Bennet et al., 2021) overlain with the TAG region (orange), and the time-varying SamCam FOV (red) and NavCam FOV (green). The red crosses that appear in the lower figure (at “Backaway” in the timeline) correspond to the thruster boresights; compare with the pre-TAG topography in Fig. 1 A and B, the thruster surface-pressure values in Fig. 10, and locations of surface erosion in Fig. 22. The right-hand movie consists of NavCam images, reprojected with North up. The red outline approximately corresponds to the SamCam FOV. Here too the red crosses that appear at “Backaway” correspond to the thruster boresight directions, and demonstrate the thrusters redirected TAGSAM ejecta visible in the NavCam (and eventually SamCam) FOVs. The SamCam and NavCam acquisition times are not simultaneous with each other, though for this sequence are generally within one second of each other. The relative display of the SamCam and NavCam image sets (i.e. when the next frame appears in the respective sequence for each camera) is correct. (MOV 5.7 MB)

(DOCX 1.5 MB)

## Data Availability

The SamCam and NavCam images used in this work are available in the OCAMS and TAGCAMS data bundles in the Planetary Data System (PDS) at https://sbn.psi.edu/pds/resource/orex/, where the instrument and UTC times of the images are effectively the image IDs.
