# Supplementary material for: Mobilization of Unconsolidated Granular Material on Asteroid (101955) Bennu by Spacecraft Interaction
Source: Space Sci Rev. 2026 Apr 1;222(3):33. doi: 10.1007/s11214-026-01285-8 (PMC13043605; doi:10.1007/s11214-026-01285-8)
Supplement: Supplementary file 2 — (DOCX 1.5 MB) [file 11214_2026_1285_MOESM2_ESM.docx]

Supplemental Information:

Mobilization of unconsolidated granular material on asteroid (101955) Bennu by spacecraft interaction

**Authors:**

Edward B. Bierhaus*^1^, Jarvis T. Songer^1^, Courtney E. Mario^2^, Christopher D. Norman^1^, Curtis Miller^1^, Ryan Olds^1^, Angelica Martinez^1^, Benton C. Clark^1,3^, Christine Hartzell^4^, Bashar Rizk^5^, Christian Drouet d’Aubigny^5^, Jennifer Nolau^6^, Alicia Allen^6^, Maurizio Pajola^7^, Dathon R Golish^5^, Humberto Campins^6^, Kevin J. Walsh^8^, Ronald-Louis Ballouz^9^, C.W.V. Wolner^5^, Brent J. Bos^10^, Dante S. Lauretta^5^, Michael C. Nolan^5^, Daniella N. DellaGiustina^5^

**Affiliations:**

^1^Lockheed Martin Space, Littleton, CO, USA.

^2^Draper, Cambridge, MA, USA.

^3^Space Science Institute, Boulder, CO, USA.

^4^University of Maryland, College Park, MD, USA.

^5^Lunar and Planetary Laboratory, University of Arizona, Tucson, AZ, USA.

^6^University of Central Florida, Orlando, FL, USA.

^7^ INAF – Astronomical Observatory of Padova, Padova, Italy.

^8^Southwest Research Institute, Boulder, CO, USA.

^9^John Hopkins University Applied Physics Laboratory, Laurel, MD, USA.

^10^NASA Goddard Spaceflight Center, Greenbelt, MD, USA.

*corresponding author: [edward.b.bierhaus@lmco.com](mailto:edward.b.bierhaus@lmco.com)

# S1. Derivation of particle dynamics observed in NavCam images

Our analysis involved measurements from images, knowledge of the spacecraft state, the Sun’s position, and Bennu’s surface (Figure S1). Specifically, three fiducials were critical to our analysis: an estimate of the NavCam position and orientation at each image derived from the spacecraft backaway attitude and trajectory, a ~5 cm/pixel DTM of the pre-contact surface, and knowledge of the Sun angle relative to the surface. We used these fiducials to make direct image measurements of the shadow motion cast by the moving ejecta plume and to solve for particle positions and velocities.


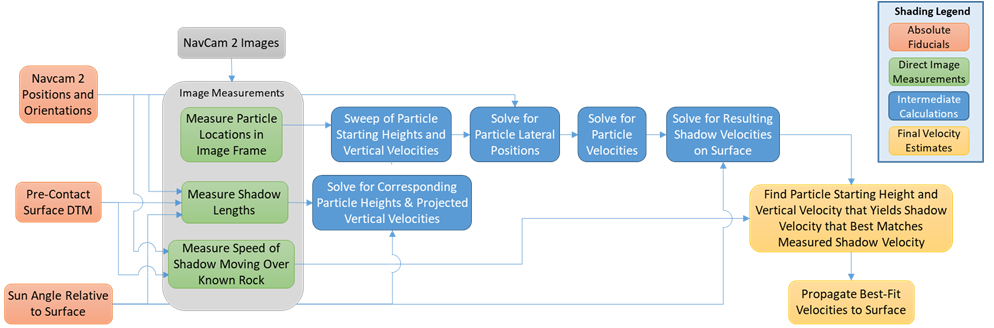


Figure S1. Flow diagram of the particle velocity estimation process.

Due to the surface disturbance after TAG and the fact that most reference features were no longer recognizable, a reconstructed trajectory of camera positions and orientations for the backaway images linked images from late in the NavCam sequence to the TAG point. All undisturbed features available for a trajectory measurement were visible in the last several backaway images. As a result, the navigation team matched as many features as possible in the later backaway images and then used the dynamics model to fit a trajectory between those images and the known TAG contact point. Our analysis used the reconstructed spacecraft trajectory, along with knowledge of the NavCam mounting on the spacecraft, to solve for the NavCam position and orientation of each image.

In the NavCam backaway images there is a rock that remains undisturbed during TAG, and is in the ground-track of the ejected material’s shadow. In other words, the shadow cast by the ejected material moves across this rock in the NavCam images (Figure S2). This shadow motion provides an estimate of the ejecta plume velocity. In the pre-contact image (Figure S2A), the rock measures 106 pixels across the dimension in the direction of the shadow motion; for the 0.0074 m/pixel of the image, resulting in the shadow traveling 0.784 m across the rock. The time between the backaway image when the shadow first touches the front of the rock to the image when the rock is completely covered in shadow is 11 s. Combining these data points results in an ejecta plume velocity measurement of 7.1 cm/s. This velocity measurement represents a projected surface shadow velocity and is dependent on both the particle motion as well as the sun angle at that image time.


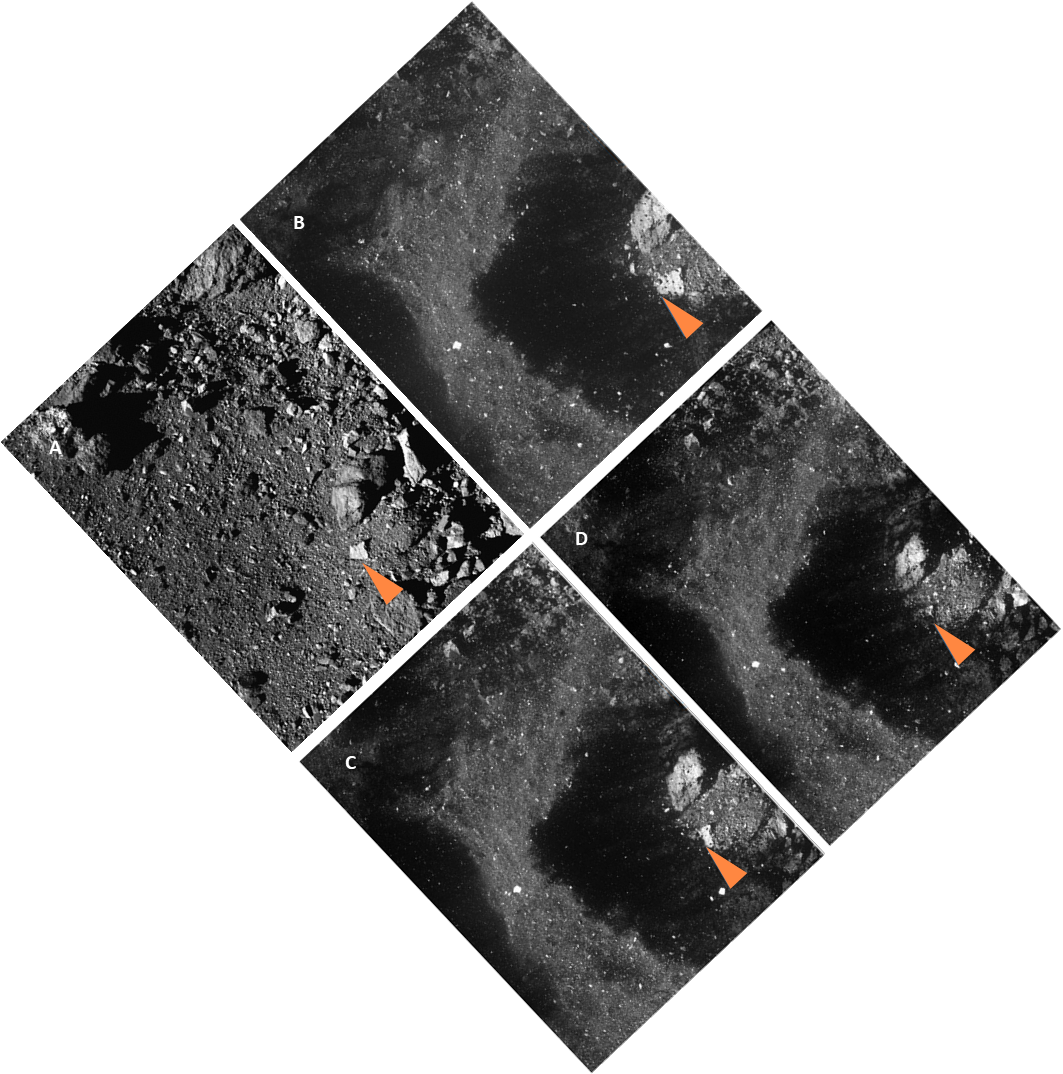


Figure S2. Shadow motion from the ejecta plume seen in backaway images. The images are not formally projected, though are rotated so that north is approximately up. For scale, the long dimension of the arrowed rock is ~0.8 m. **(A)** The surface prior to contact, where the orange arrow points to a rock that is distinguishable in the backaway images and can be used as a reference to estimate plume velocity. (**B** to **D**) Motion of the shadow cast by the plume as the shadow moves across this rock, with the orange arrow highlighting the leading edge of the shadow.

The shadow also provides insight into ejecta-plume height, which can be estimated from the measured length of the shadow in the backaway images and knowledge of the Sun angle relative to the surface at that time. The shadow length was measured along lines parallel to the Sun direction (Figure S3). This process was performed for backaway image 96 since that is one of the first images where the shadow could easily be identified. It was then repeated for image 131, one of the last NavCam images acquired, as well as two intermediate images.


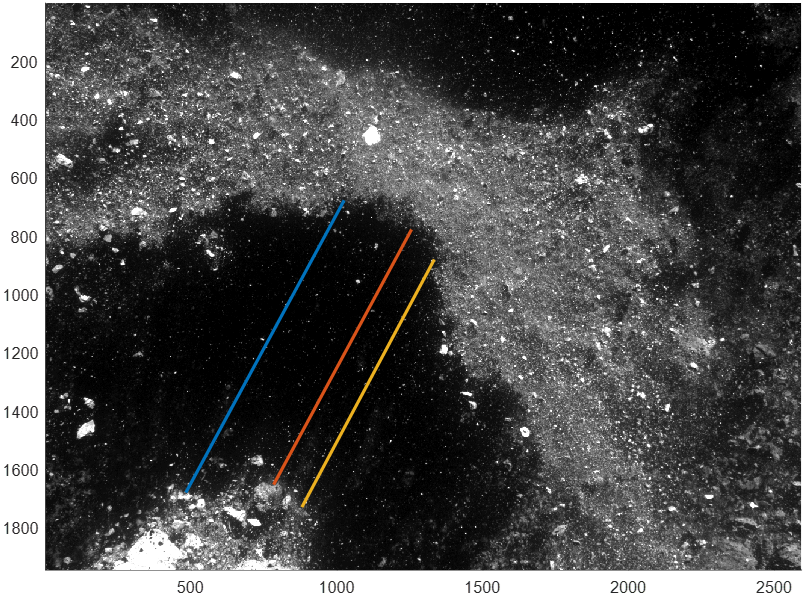


Figure S3. Example lines (image 96) used for shadow length estimate.

We converted these pixel shadow measurements to a physical distance using the estimated spacecraft position and orientation at the time of the image, the pre-contact DTM of the surface, and the Sun angle relative to the surface. The spacecraft position and orientation were used to project the image pixel locations for the end of each shadow line onto the surface of the pre-contact DTM. Due to underlying surface height variation and the fact that the leading edge of the shadow crosses over surface terrain 0.5 to 2 m above the shadow origin locations, the horizontal shadow length, l_shadow_, for the shadow measured between points p_1_ and p_2_ was computed as follows:

| $l_{meas}= \sqrt{\sum\left( p_{1}\left( x,y \right)-p_{2}(x,y) \right)^{2}}$ | (S1) |
| --- | --- |

| $l_{shadow}=l_{meas}+\frac{\left( p_{2}\left( z \right)-p_{1}(z) \right)}{tan(\theta_{sun,el})}$ | (S2) |
| --- | --- |

where $\theta_{sun,el}$ is the sun elevation angle relative to the surface. This calculation therefore provides a measure of the horizontal shadow length if the surface were perfectly smooth and using the sun elevation angle can be used to calculate the corresponding heights of the particles that cast the shadows. Figure S4 shows the results of this calculation for multiple shadow lengths in 4 different images. Fitting a line to these heights yields an average projected velocity of 6.3 cm/s; if the ejected material were moving only vertically, this projected magnitude would be the maximum possible vertical speed.

Figure S4. Particle heights corresponding to measured shadow lengths. If the particles are traveling only vertically, these heights would correspond to an average vertical velocity of 6.3 cm/s.

In addition to using shadow measurements to derive bulk material speeds, we derived positions and velocities of discrete particles. To measure particle positions and velocities over time, particle locations were measured in the backaway images, in image pixel coordinates, over multiple NavCam images. We used images 95 to 131 to leverage the observations from the shadow-length measurements. The significant variability in appearance of an individual particle between frames (due to particle rotation) prevented automated tracking. As a result, these tracks were defined manually at an interval of every three images. We selected particles at locations distributed across the boundary of the bulk ejecta plume that are redirected by the thrusters (Figure S5). This dataset includes 21 particles that primarily move with the ejected material that casts the large shadow, as well as three particles that move in inconsistent directions and at higher velocities.


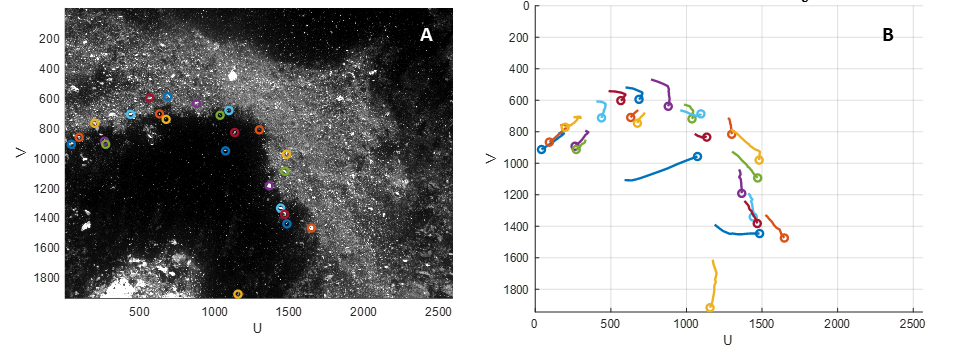


Figure S5. Tracked particles used for velocity analysis. **(A)** Tracked particles in backaway image 95 (49 seconds after initial surface contact). **(B)** Image pixel coordinates of tracked particles from image 95 to 131, where the circle represents the start of each track and corresponds to the particle locations shown in (A). Particle colors in (A) correspond to the track colors in (B).

We implemented a particle state solution that leveraged the constraints on heights and velocities estimated via shadow tracking. In an image, the relationship between how a 3D point in the asteroid centered fixed (ACF) frame projects into image is given by the following:

| $\left[ \begin{matrix} u \\ v \end{matrix} \right]=\left[ \begin{matrix} x_{cam}/z_{cam} \\ y_{cam}/z_{cam} \end{matrix} \right]$ | (S3) |
| --- | --- |

| $\left[ \begin{matrix} x_{cam} \\ y_{cam} \\ z_{cam} \end{matrix} \right] =K\left[ \begin{matrix} R & t \end{matrix} \right]\left[ \begin{matrix} x_{ACF} \\ y_{ACF} \\ \begin{matrix} z_{ACF} \\ 1 \end{matrix} \end{matrix} \right]$ | (S4) |
| --- | --- |

where, for an undistorted image with the origin defined in the top left corner, u is the pixel coordinate column number, v is the pixel coordinate row number, (x,y,z)_cam_ is the position in the camera frame, K is the camera matrix, R is the rotation from ACF to the camera frame for that image, t is the position of the camera in the ACF frame, and (x,y,z)_ACF_ is the position in the ACF frame. The ACF frame is the body-fixed frame, which is a rotating frame that rotates about the asteroid’s spin axis at the same rate as the asteroid.

To solve for a particle’s position in ACF, a single image pixel coordinate is insufficient since there are 3 unknowns with only 2 equations. Using the particle heights and vertical velocities derived as described above, we simulated particle heights in each image to define z_ACF_ and use their detected image pixel coordinate (u,v) in each image, and subsequently solve for each particle’s lateral position (x_ACF_, y_ACF_) at that height in each image. This is solved as a nonlinear least squares minimization problem to find the values of x_ACF_, and y_ACF_ that minimize the error between the measured (u,v) location of the particle in the image and the corresponding projected image pixel coordinate using the particle’s ACF location. Once an individual particle’s ACF positions are known for each image that the particle is detected in, the particle’s velocity between images can then be computed.

To apply this approach to the particles in the backaway images, simulated particle heights for each image were needed. From shadow measurements (Figure S4), we selected initial particle heights that varied from 0 to 3 m above the surface, with vertical velocities varying from –10 to 8 cm/s. While these starting heights and velocity ranges are larger than the 2 to 3 m, 6.3 cm/s average constraints, selecting a wide range of potential particle heights and vertical velocities ensured we did not overlook a potential solution. Particle heights were then simulated for images 95 to 131 for each of these cases, resulting in 1456 different vertical trajectories. Each particle’s lateral position was then solved for along these trajectories in each image that had a valid particle pixel measurement and the resulting particle positions over time were used to solve for particle velocities. All particle positions and velocities were estimated in a local TAG frame, which was defined with z pointing normal to the surface at the TAG contact location, so that the x and y velocities represented lateral velocities relative to Bennu’s surface.

The next step was to determine which of the simulated vertical trajectories resulted in particle velocities that best fit the direct particle velocity measurements from the images. For this we used the constraint derived from the projected shadow speed on the surface. We applied the particle position solutions, and the sun elevation and azimuth angles, to project where the shadow cast by each particle would intersect the surface. To simplify the approach, the surface was assumed to be a flat plane. Then, using the shadow surface intersection points, the shadow surface velocity was calculated from the lateral distance shadow traveled over time and compared with the 7.1 cm/s observed projected speed (Figure 14).

Comparing these results to the estimated 7.1 cm/s shadow velocity shows that two vertical velocities, –1 cm/s and –7 cm/s, result in similar shadow speeds. However, when plotting these shadow trajectories, the shadow projected from the –7 cm/s vertical velocity results in a shadow moving in the opposite direction from what is observed in the images; i.e., the particles are approaching to the surface fast enough that the predicted shadow height decreases. In comparison, the shadow projected from the –1 cm/s vertical velocity matches the magnitude and direction of the projected surface shadow seen in the images. We used the best-fit projected shadow speed and vertical speed to constrain the height of the particles in the initial NavCam frame used, and found that a ~ 2 m height best fits the observed shadow geometry.

# S2. Estimated erosion and derivation of revised mass flux

In this section we provide (1) the basis for numerical values used in Equation 6 that produced Figure 23 A and C, and (2) a quantitative though simplified treatment of a complex physical system to derive an alternate version of the surface erosion. Related literature (including, but not limited to, multiple Rajaratnam et al., Metzger et al. LaMarche 2013, Morris et al. papers) demonstrate that details, such as those related to plume evolution over time and two-phase flow, affect the results. Our objective was to derive an estimate for the mass flux that approximated the observed values, using basic conservation relationships.

S2.1 Derivation of numerical values for Equation 6

To develop quantified inputs for Equation 6 we used of mix of data directly from our simulations, and calculations that leveraged our simulation data, plus other parameters related to Bennu and the thruster gas. In particular, we followed Metzger (2024a) and (2024b) where we could, and developed functionally-equivalent values where we could not implement similar derivations:

- Our simulations provide values for *ρ*_0_, *v*_0_, and gas temperature *T*. We extracted gas plume quantities from the simulation mesh at a height <D> above each surface element; we derive <D> below.
- Gas temperature enabled us to derive a value for $\bar{v_{T}}$, where $\bar{v_{T}}\sim1.6 \sqrt{k_{B}T/m}$, for which *k*_B_ is Boltzmann’s constant, and *m* is the mass of a gas molecule. We estimated 12.6 g/mol for the weighted average molar mass of the thruster gas.
- *ρ*_b_ ~ 1200 kg/m^3^ (Scheeres et al. 2019)
- *g* = 5.95 × 10^-5^ m/s^2^ (Daly et al. 2020a)
- To estimate <D> we considered the particle size-frequency distribution (SFD) for Nightingale from Burke et al. (2021). They show the SFD is in equilibrium at sizes between ~5 cm (the completeness limit) and ~30 cm. Equilibrium corresponds to a differential power-law slope of about -3. In this scenario, smaller particles dominate the surface area (e.g. Bierhaus et al. 2023). Also, in a negatively-sloped power law, the average particle diameter is much closer to the minimum size in the population than the maximum size. Thus, within the accuracy of the data, 5 cm is the minimum diameter particle, larger than the observable completeness limit, with the maximum surface area in the region. To add some conservatism (i.e. avoid underestimating the maximum possible average particle size), we use 10 cm (0.1 m) rather than 5 cm.
- For cohesion, we use 0.001 Pa based on our analysis described in the previous section.
- The two parameters that are the most difficult to derive as done in the Metzger (2024a) and (2024b) papers are *E*_th_, and ε.
  - For *E*_th_, we (i) leverage the concept behind the parameter, i.e. it is the energy flux threshold at which erosion starts, in conjunction with (ii) the threshold shear velocity from Kok et al. (2012), which is the velocity at which wind mobilizes a stationary particle, see their equation 2.8. This equation includes two empirical parameters, which Kok et al. provide for dry, loose dust, and thus we adopt their values. We use this velocity as $v_{*}$ in *E*_th_ = $\rho_{0}v_{*}^{2}\bar{v_{T}}/12$. Note that because *E*_th_ depends on both *ρ*_0_ and *v*, we calculate a unique value for each facet. Our median value for the backaway start simulation is 0.01 J/m^2^/s, compared with the Metzger (2024b) value of 0.123 J/m^2^/s for the Moon.
  - Metzger (2024b) derives ε by evaluating Apollo descent video, determining when dust mobilization starts, and estimating the amount of dust. We cannot employ a similar technique. For the purposes of this basic comparison, we adopt the Metzger (2024b) value of 0.0029. This is reasonable given (i) the parameter is the fraction of the plume energy that contributes to erosion, and (ii) the high porosity of the immediate upper few cm of the lunar regolith, as well as the upper ~10 cm of the Bennu regolith.

The erosion depths estimated by this technique significantly exceed the observed values (Figure 23). Before deriving an alternative formulation, we first considered whether any of the numerical values we used as inputs had an allowable range that would reduce the estimated erosion rate by the orders of magnitude necessary to be consistent with observations:

- ε: This value would need to decrease to drive $\dot{m}$ lower. Porosity is apparently an important factor in this parameter – i.e. how easy is it for the thruster plume to penetrate the regolith. Even though the lunar regolith is not very porous below several cm, the upper few cm is quite porous. We know Bennu has a porous, ~10 cm upper layer too (Walsh et al 2022, and results from analysis reported in this paper). Thus it seems that this parameter could be similar between the two bodies; if different on Bennu it is most likely higher (Bennu’s relatively high porosity goes deeper than on the Moon).
- *E_th_*: This value would need to increase to drive $\dot{m}$ lower. This is unlikely given our value is approximately six times smaller than the Moon, and Bennu’s gravity and cohesion are orders of magnitude lower than the Moon.
  - Metzger (2024a) notes that saltation, the process of a dust grain impacting a granular bed and mobilizing additional grains, does not apply in the case of deriving the magnitude of thruster-driven erosion because the low gravity and vacuum means an ejected particle lands far from the ejection point. While saltation, derived from studies of wind-driven particle erosion on planetary surfaces with atmospheres, does not apply to the plume impingement region, we expect the underlying initiation of the first mobilized particles is relevant. A smaller value of *E_th_* at Bennu makes sense because this parameter is related to the minimum energy needed to mobilize a particle, which depends on gravity and cohesion. In addition, only ~0.3% of facets in the simulation domain have *E* < *E_th_*, thus even if the actual value of *E_th_* is much smaller, the practical effect of the current calculation is negligible because only a few facets would have decreased $\dot{m}$.
- *g*: This value would need to increase for $\dot{m}$ to go down. An unusual feature of small bodies is that the surface acceleration due to gravity is so low that the surface rotational acceleration can have a meaningful effect on the net surface acceleration value as a function of latitude (Scheeres et al. 2010). However, the net surface acceleration of Bennu only varies by ~38% across the surface (Daly et al. 2020a).
- <D> = This value would need to increase for $\dot{m}$ to go down. The particle size here may be in part a proxy for surface roughness, which defines the thickness of gas flow that could be turbulent due to small-scale surface roughness. In this interpretation, of-order a few cm is an appropriate value. There are larger particles with larger heights across the TAG region, perhaps this value could increase by a factor of a few. Surface roughness is related to, but not the same as particle size. Thus if this parameter is more related to the true abundance of particles of certain sizes, then the minimum value could drop to a few mm. However, reducing <D> would *increase* $\dot{m}$.
- α: This value would need to increase for $\dot{m}$ to go down. Several studies show Bennu’s cohesion is quite low, no more than a few Pa, and our own analysis (Figure 20) demonstrates the cohesion is < 0.1 Pa. In addition, it is possible that the effect of cohesion is “double counted” in: the *E_th_* term in the numerator already establishes that the plume energy must exceed some threshold before the particle moves, presumably that threshold includes cohesion, and even inertial forces.

We considered a case with the parameters changed to minimize $\dot{m}$ in which we increased <D> to 0.3 m and α to 0.1 Pa, and the resulting median erosional depth was ~1.2 m, with 20% of facets eroding more than 8 m. While this is closer to observations, it is nevertheless still more than an order of magnitude too large.

Thus we were motivated to consider an alternate formulation of Equation 6 that reduces the efficiency of the thruster erosion. Fundamentally the logic of $\dot{m}$ as formulated Metzger (2024a) and (2024b) is robust: essentially it is the ratio of the capability of the plume to move material to the material’s resistance to motion. The basic condition that the plume must exceed some threshold of resistance should be true. However, we noted that the existing formulation does not deduct the kinetic energy of regolith from the kinetic energy in the plume. The two terms in the denominator of Equation 6 account for the plume forces counter-acting gravity and cohesion but they do not assign any plume kinetic energy to changing a surface particle’s speed from zero to a non-zero value. Yet it must be the case that any speed achieved by a particle mobilized by the thrusters is sourced from thruster kinetic energy. Put another way, the Metzger (2024a) and (2024b) formulation assumes that mass loading does not affect plume erosion efficiency. To address this transfer of energy from the plume to the regolith we considered an additional factor that relates to mass loading in the plume (Figure S6). While the assumption that mass loading from mobilized regolith likely is the case under certain conditions, perhaps this assumption does not apply in the case when there is a significant amount of mass mobilized, which is the case for the OSIRIS-REx TAG event.


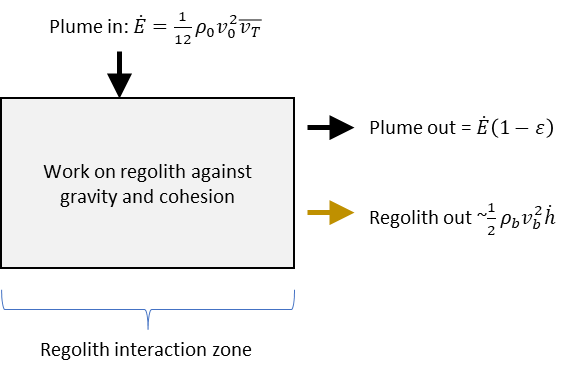


Figure S6. Control volume for revised erosion calculation.

We derived a simplified yet general approach to approximate this effect as follows:

First, recast $\dot{m}$ as:

| $\dot{m}=\dot{h}\rho_{b}=\rho_{b}\frac{\varepsilon\left( {\frac{1}{12}\rho}_{0}v_{0}^{2}\bar{v_{T}}-E_{th} \right)}{\rho_{b}g\left\langle D \right\rangle+\alpha}$ | (S5) |
| --- | --- |

where $\dot{h}$ is an erosion rate in m/s. Rearranging and adding a term, $\frac{1}{2}\rho_{b}{\bar{v_{b}}}^{2}$*,* to account for the kinetic energy of the regolith results in:

| $\dot{h}\left( \rho_{b}g\left\langle D \right\rangle+\alpha+\frac{1}{2}\rho_{b}{\bar{v_{b}}}^{2} \right)=\varepsilon\left( {\frac{1}{12}\rho}_{0}v_{0}^{2}\bar{v_{T}}-E_{th} \right)$ | (S6) |
| --- | --- |

where $\bar{v_{b}}$ is the average velocity imparted to the regolith excavated by the thruster plumes, and which depends on local conditions. We expect the value is related to drag forces on particles and thus varies throughout the impingement zone, in which case:

| $\rho_{b}{\bar{v_{b}}}^{2}\propto\rho_{0}v_{0}^{2}$ | (S7) |
| --- | --- |

Assuming $\rho_{b}$ is constant, then:

| ${\frac{1}{2}\rho}_{b}{\bar{v_{b}}}^{2}=c \rho_{0}v_{0}^{2}$ | (S8) |
| --- | --- |

where *c* is a constant derived via correlation or particle+flowfield analysis. Now substitute Equation S8 into S6, and solve for $\dot{h}:$

| $\dot{h}=\frac{\varepsilon\left( {\frac{1}{12}\rho}_{0}v_{0}^{2}\bar{v_{T}}-E_{th} \right)}{\rho_{b}g\left\langle D \right\rangle+\alpha+c \rho_{0}v_{0}^{2}}$ | (S9) |
| --- | --- |

Equation $\dot{h}$ provides an erosion rate in m/s that accounts for the plume energy lost to both releasing a particle from the surface (the $\rho_{b}g\left\langle D \right\rangle+\alpha$ term in the denominator) as well as the addition of kinetic energy imparted to that material (the $\rho_{0}v_{0}^{2}$term in the denominator).

# S3. CAPTION FOR MOVIE S1

**Movie generated from SamCam and NavCam image data.** The graphic in the upper left is the timeline of the TAG event. The moving red vertical line in the timeline corresponds to the time of each image in the movie. The labeled events in the timeline include: “TAG” for first contact, “Gas” for TAGSAM sample gas release, and “Backaway” for the start of the backaway thrusters. The thick black arrows indicate the directions for Bennu North (N) and the Sun. The left-hand portion of the movie consists of SamCam images, showing TAGSAM, the TAGSAM arm, and Bennu’s surface. The center portion of the movie consists of static images extracted from the Bennu basemap (Bennet et al. 2021) overlain with the TAG region (orange), and the time-varying SamCam FOV (red) and NavCam FOV (green). The red crosses that appear in the lower figure (at “Backaway” in the timeline) correspond to the thruster boresights; compare with the pre-TAG topography in Figure 1 A and B, the thruster surface-pressure values in Figure 10, and locations of surface erosion in Figure 17. The right-hand portion of the movie consists of NavCam images, reprojected with North up. The red outline approximately corresponds to the SamCam FOV. Here too the red crosses that appear at “Backaway” correspond to the thruster boresight directions, and demonstrate the thrusters redirected TAGSAM ejecta visible in the NavCam (and eventually SamCam) FOVs. The SamCam and NavCam acquisition times are not simultaneous with each other, though for this sequence are generally within one second of each other. The relative display of the SamCam and NavCam image sets (i.e. when the next frame appears in the respective sequence for each camera) is correct.
